# Supplementary material for: Advancements in genetic research by the Hispanic Community Health Study/Study of Latinos: A 10-year retrospective review
Source: HGG Adv. 2024 Oct 29;6(1):100376. doi: 10.1016/j.xhgg.2024.100376 (PMC11754138; doi:10.1016/j.xhgg.2024.100376)
Supplement: Document S1. Table S1 [file mmc1.pdf]

HGGA, Volume *6*

Supplemental information

Advancements in genetic research by the Hispanic Community Health Study/Study of Latinos: A 10-year retrospective review

Hridya Rao, Margaret C. Weiss, Jee Young Moon, Krista M. Perreira, Martha L. Daviglus, Robert Kaplan, Kari E. North, Maria Argos, Lindsay Fernández-Rhodes, and Tamar Sofer

Supplementary Table

Table S1: Summary of genetic epidemiological studies for health outcomes in HCHS/SOL

Study type	Health outcome or trait
Genome wide association studies	Metabolome (1)<br>Blood traits (2-6)<br>Pulmonary disease and function (7-9)<br>Cardiometabolic traits (10-15)<br>Cardiovascular conduction (16-20)<br>Kidney disease and function (21)<br>Cognitive function, behavior, depression, and anxiety (22-26)<br>Dental conditions (27-30)
Admixture mapping	Cardiometabolic traits (31, 32)<br>Kidney traits (33, 34)<br>Pulmonary function (7, 35)
Polygenic risk score	Anthropometrics and obesity (36-41)<br>Cardiometabolic traits (37, 42-44)<br>Cognition and psychiatric traits (22, 23)<br>Pulmonary function (37, 42, 45, 46)
Fine mapping	Anthropometrics (47, 48)<br>Cardiometabolic traits (18, 43, 49-53)<br>Kidney function (54)<br>Reproductive traits (55)<br>Blood traits (56, 57)
Mendelian Randomization	Cardiometabolic traits (58)<br>Pulmonary function (42, 59, 60)<br>Cognition (61)
Gene by Environment Interaction	Anthropometrics and obesity (36, 38)<br>Cardiometabolic traits (62, 63)<br>Cognition and psychiatric traits (64)
Metabolomics integration	Cardiometabolic traits (1, 65-67)<br>Cognition and sleep (68, 69)<br>Macular Degeneration (70)

Supplementary references

1. Feofanova EV, Chen H, Dai Y, Jia P, Grove ML, Morrison AC, et al. A Genome-wide Association Study Discovers 46 Loci of the Human Metabolome in the Hispanic Community Health Study/Study of Latinos. Am J Hum Genet. 2020;107(5):849-63.
2. Moon JY, Louie TL, Jain D, Sofer T, Schurmann C, Below JE, et al. A Genome-Wide Association Study Identifies Blood Disorder-Related Variants Influencing Hemoglobin A(1c) With Implications for Glycemic Status in U.S. Hispanics/Latinos. Diabetes Care. 2019;42(9):1784-91.
3. Jain D, Hodonsky CJ, Schick UM, Morrison JV, Minnerath S, Brown L, et al. Genome-wide association of white blood cell counts in Hispanic/Latino Americans: the Hispanic Community Health Study/Study of Latinos. Hum Mol Genet. 2017;26(6):1193-204.
4. Raffield LM, Louie T, Sofer T, Jain D, Ipp E, Taylor KD, et al. Genome-wide association study of iron traits and relation to diabetes in the Hispanic Community Health Study/Study of Latinos (HCHS/SOL): potential genomic intersection of iron and glucose regulation? Hum Mol Genet. 2017;26(10):1966-78.
5. Schick UM, Jain D, Hodonsky CJ, Morrison JV, Davis JP, Brown L, et al. Genome-wide Association Study of Platelet Count Identifies Ancestry-Specific Loci in Hispanic/Latino Americans. Am J Hum Genet. 2016;98(2):229-42.
6. Hodonsky CJ, Jain D, Schick UM, Morrison JV, Brown L, McHugh CP, et al. Genome-wide association study of red blood cell traits in Hispanics/Latinos: The Hispanic Community Health Study/Study of Latinos. PLoS Genet. 2017;13(4):e1006760.
7. Burkart KM, Sofer T, London SJ, Manichaikul A, Hartwig FP, Yan Q, et al. A Genome-Wide Association Study in Hispanics/Latinos Identifies Novel Signals for Lung Function. The Hispanic Community Health Study/Study of Latinos. Am J Respir Crit Care Med. 2018;198(2):208-19.
8. Yan Q, Brehm J, Pino-Yanes M, Forno E, Lin J, Oh SS, et al. A meta-analysis of genome-wide association studies of asthma in Puerto Ricans. Eur Respir J. 2017;49(5).
9. Zhao X, Qiao D, Yang C, Kasela S, Kim W, Ma Y, et al. Whole genome sequence analysis of pulmonary function and COPD in 19,996 multi-ethnic participants. Nat Commun. 2020;11(1):5182.
10. Sofer T, Wong Q, Hartwig FP, Taylor K, Warren HR, Evangelou E, et al. Genome-Wide Association Study of Blood Pressure Traits by Hispanic/Latino Background: the Hispanic Community Health Study/Study of Latinos. Sci Rep. 2017;7(1):10348.
11. Justice AE, Young K, Gogarten SM, Sofer T, Graff M, Love SAM, et al. Genome-wide association study of body fat distribution traits in Hispanics/Latinos from the HCHS/SOL. Hum Mol Genet. 2021;30(22):2190-204.
12. Qi Q, Stilp AM, Sofer T, Moon JY, Hidalgo B, Szpiro AA, et al. Genetics of Type 2 Diabetes in U.S. Hispanic/Latino Individuals: Results From the Hispanic Community Health Study/Study of Latinos (HCHS/SOL). Diabetes. 2017;66(5):1419-25.
13. Sung YJ, Winkler TW, de Las Fuentes L, Bentley AR, Brown MR, Kraja AT, et al. A Large-Scale Multi-ancestry Genome-wide Study Accounting for Smoking Behavior Identifies Multiple Significant Loci for Blood Pressure. Am J Hum Genet. 2018;102(3):375-400.
14. Liang J, Le TH, Edwards DRV, Tayo BO, Gaulton KJ, Smith JA, et al. Single-trait and multi-trait genome-wide association analyses identify novel loci for blood pressure in African-ancestry populations. PLoS Genet. 2017;13(5):e1006728.
15. Sofer T, Emery L, Jain D, Ellis AM, Laurie CC, Allison MA, et al. Variants Associated with the Ankle Brachial Index Differ by Hispanic/Latino Ethnic Group: a genome-wide

association study in the Hispanic Community Health Study/Study of Latinos. Sci Rep. 2019;9(1):11410.
16. Napier MD, Franceschini N, Gondalia R, Stewart JD, Mendez-Giraldez R, Sitlani CM, et al. Genome-wide association study and meta-analysis identify loci associated with ventricular and supraventricular ectopy. Sci Rep. 2018;8(1):5675.
17. Kerr KF, Avery CL, Lin HJ, Raffield LM, Zhang QS, Browning BL, et al. Genome-wide association study of heart rate and its variability in Hispanic/Latino cohorts. Heart Rhythm. 2017;14(11):1675-84.
18. Seyerle AA, Lin HJ, Gogarten SM, Stilp A, Mendez Giraldez R, Soliman E, et al. Genome-wide association study of PR interval in Hispanics/Latinos identifies novel locus at ID2. Heart. 2018;104(11):904-11.
19. Swenson BR, Louie T, Lin HJ, Mendez-Giraldez R, Below JE, Laurie CC, et al. GWAS of QRS duration identifies new loci specific to Hispanic/Latino populations. PLoS One. 2019;14(6):e0217796.
20. Mendez-Giraldez R, Gogarten SM, Below JE, Yao J, Seyerle AA, Highland HM, et al. GWAS of the electrocardiographic QT interval in Hispanics/Latinos generalizes previously identified loci and identifies population-specific signals. Sci Rep. 2017;7(1):17075.
21. Qian H, Kowalski MH, Kramer HJ, Tao R, Lash JP, Stilp AM, et al. Genome-Wide Association of Kidney Traits in Hispanics/Latinos Using Dense Imputed Whole-Genome Sequencing Data: The Hispanic Community Health Study/Study of Latinos. Circ Genom Precis Med. 2020;13(4):e002891.
22. Jian X, Sofer T, Tarraf W, Bressler J, Faul JD, Zhao W, et al. Genome-wide association study of cognitive function in diverse Hispanics/Latinos: results from the Hispanic Community Health Study/Study of Latinos. Transl Psychiatry. 2020;10(1):245.
23. Dunn EC, Sofer T, Wang MJ, Soare TW, Gallo LC, Gogarten SM, et al. Genome-wide association study of depressive symptoms in the Hispanic Community Health Study/Study of Latinos. J Psychiatr Res. 2018;99:167-76.
24. Dunn EC, Sofer T, Gallo LC, Gogarten SM, Kerr KF, Chen CY, et al. Genome-wide association study of generalized anxiety symptoms in the Hispanic Community Health Study/Study of Latinos. Am J Med Genet B Neuropsychiatr Genet. 2017;174(2):132-43.
25. Saccone NL, Emery LS, Sofer T, Gogarten SM, Becker DM, Bottinger EP, et al. Genome-Wide Association Study of Heavy Smoking and Daily/Nondaily Smoking in the Hispanic Community Health Study/Study of Latinos (HCHS/SOL). Nicotine Tob Res. 2018;20(4):448-57.
26. Khoury S, Wang QP, Parisien M, Gris P, Bortsov AV, Linnstaedt SD, et al. Multi-ethnic GWAS and meta-analysis of sleep quality identify MPP6 as a novel gene that functions in sleep center neurons. Sleep. 2021;44(3).
27. Sanders AE, Sofer T, Wong Q, Kerr KF, Agler C, Shaffer JR, et al. Chronic Periodontitis Genome-wide Association Study in the Hispanic Community Health Study / Study of Latinos. J Dent Res. 2017;96(1):64-72.
28. Shungin D, Haworth S, Divaris K, Agler CS, Kamatani Y, Keun Lee M, et al. Genome-wide analysis of dental caries and periodontitis combining clinical and self-reported data. Nat Commun. 2019;10(1):2773.
29. Morrison J, Laurie CC, Marazita ML, Sanders AE, Offenbacher S, Salazar CR, et al. Genome-wide association study of dental caries in the Hispanic Communities Health Study/Study of Latinos (HCHS/SOL). Hum Mol Genet. 2016;25(4):807-16.
30. Sanders AE, Jain D, Sofer T, Kerr KF, Laurie CC, Shaffer JR, et al. GWAS Identifies New Loci for Painful Temporomandibular Disorder: Hispanic Community Health Study/Study of Latinos. J Dent Res. 2017;96(3):277-84.

31. Sofer T, Baier LJ, Browning SR, Thornton TA, Talavera GA, Wassertheil-Smoller S, et al. Admixture mapping in the Hispanic Community Health Study/Study of Latinos reveals regions of genetic associations with blood pressure traits. PLoS One. 2017;12(11):e0188400.
32. Andaleon A, Mogil LS, Wheeler HE. Genetically regulated gene expression underlies lipid traits in Hispanic cohorts. PLoS One. 2019;14(8):e0220827.
33. Brown LA, Sofer T, Stilp AM, Baier LJ, Kramer HJ, Masindova I, et al. Admixture Mapping Identifies an Amerindian Ancestry Locus Associated with Albuminuria in Hispanics in the United States. J Am Soc Nephrol. 2017;28(7):2211-20.
34. Horimoto A, Xue D, Cai J, Lash JP, Daviglus ML, Franceschini N, et al. Genome-Wide Admixture Mapping of Estimated Glomerular Filtration Rate and Chronic Kidney Disease Identifies European and African Ancestry-of-Origin Loci in Hispanic and Latino Individuals in the United States. J Am Soc Nephrol. 2022;33(1):77-87.
35. Wang H, Cade BE, Sofer T, Sands SA, Chen H, Browning SR, et al. Admixture mapping identifies novel loci for obstructive sleep apnea in Hispanic/Latino Americans. Hum Mol Genet. 2019;28(4):675-87.
36. Moon JY, Wang T, Sofer T, North KE, Isasi CR, Cai J, et al. Objectively Measured Physical Activity, Sedentary Behavior, and Genetic Predisposition to Obesity in U.S. Hispanics/Latinos: Results From the Hispanic Community Health Study/Study of Latinos (HCHS/SOL). Diabetes. 2017;66(12):3001-12.
37. Sofer T, Moon JY, Isasi CR, Qi Q, Shah NA, Kaplan RC, et al. Relationship of genetic determinants of height with cardiometabolic and pulmonary traits in the Hispanic Community Health Study/Study of Latinos. Int J Epidemiol. 2018;47(6):2059-69.
38. McArdle CE, Bokhari H, Rodell CC, Buchanan V, Preudhomme LK, Isasi CR, et al. Findings from the Hispanic Community Health Study/Study of Latinos on the Importance of Sociocultural Environmental Interactors: Polygenic Risk Score-by-Immigration and Dietary Interactions. Front Genet. 2021;12:720750.
39. Isasi CR, Moon JY, Gallo LC, Qi Q, Wang T, Sotres-Alvarez D, et al. Chronic Stress, Genetic Risk, and Obesity in US Hispanic/Latinos: Results From the Hispanic Community Health Study/Study of Latinos. Psychosom Med. 2022;84(7):822-7.
40. Grinde KE, Qi Q, Thornton TA, Liu S, Shadyab AH, Chan KHK, et al. Generalizing polygenic risk scores from Europeans to Hispanics/Latinos. Genet Epidemiol. 2019;43(1):50-62.
41. Spear ML, Diaz-Papkovich A, Ziv E, Yracheta JM, Gravel S, Torgerson DG, et al. Recent shifts in the genomic ancestry of Mexican Americans may alter the genetic architecture of biomedical traits. Elife. 2020;9.
42. Zhang Y, Elgart M, Kurniansyah N, Spitzer BW, Wang H, Kim D, et al. Genetic determinants of cardiometabolic and pulmonary phenotypes and obstructive sleep apnoea in HCHS/SOL. EBioMedicine. 2022;84:104288.
43. Hu Y, Graff M, Haessler J, Buyske S, Bien SA, Tao R, et al. Minority-centric meta-analyses of blood lipid levels identify novel loci in the Population Architecture using Genomics and Epidemiology (PAGE) study. PLoS Genet. 2020;16(3):e1008684.
44. Kurniansyah N, Goodman MO, Kelly TN, Elfassy T, Wiggins KL, Bis JC, et al. A multi-ethnic polygenic risk score is associated with hypertension prevalence and progression throughout adulthood. Nat Commun. 2022;13(1):3549.
45. Guo Y, Moon JY, Laurie CC, North KE, Sanchez-Johnsen LAP, Davis S, et al. Genetic predisposition to obesity is associated with asthma in US Hispanics/Latinos: Results from the Hispanic Community Health Study/Study of Latinos. Allergy. 2018;73(7):1547-50.
46. Hu X, Qiao D, Kim W, Moll M, Balte PP, Lange LA, et al. Polygenic transcriptome risk scores for COPD and lung function improve cross-ethnic portability of prediction in the NHLBI TOPMed program. Am J Hum Genet. 2022;109(5):857-70.
47. Fernandez-Rhodes L, Gong J, Haessler J, Franceschini N, Graff M, Nishimura KK, et al. Trans-ethnic fine-mapping of genetic loci for body mass index in the diverse ancestral

populations of the Population Architecture using Genomics and Epidemiology (PAGE) Study reveals evidence for multiple signals at established loci. Hum Genet. 2017;136(6):771-800.
48. Fernandez-Rhodes L, Graff M, Buchanan VL, Justice AE, Highland HM, Guo X, et al. Ancestral diversity improves discovery and fine-mapping of genetic loci for anthropometric traits-The Hispanic/Latino Anthropometry Consortium. HGG Adv. 2022;3(2):100099.
49. Zubair N, Graff M, Luis Ambite J, Bush WS, Kichaev G, Lu Y, et al. Fine-mapping of lipid regions in global populations discovers ethnic-specific signals and refines previously identified lipid loci. Hum Mol Genet. 2016;25(24):5500-12.
50. Franceschini N, Carty CL, Lu Y, Tao R, Sung YJ, Manichaikul A, et al. Variant Discovery and Fine Mapping of Genetic Loci Associated with Blood Pressure Traits in Hispanics and African Americans. PLoS One. 2016;11(10):e0164132.
51. Bien SA, Pankow JS, Haessler J, Lu Y, Pankratz N, Rohde RR, et al. Transethnic insight into the genetics of glycaemic traits: fine-mapping results from the Population Architecture using Genomics and Epidemiology (PAGE) consortium. Diabetologia. 2017;60(12):2384-98.
52. Kocarnik JM, Richard M, Graff M, Haessler J, Bien S, Carlson C, et al. Discovery, fine-mapping, and conditional analyses of genetic variants associated with C-reactive protein in multiethnic populations using the Metabochip in the Population Architecture using Genomics and Epidemiology (PAGE) study. Hum Mol Genet. 2018;27(16):2940-53.
53. Downie CG, Dimos SF, Bien SA, Hu Y, Darst BF, Polfus LM, et al. Multi-ethnic GWAS and fine-mapping of glycaemic traits identify novel loci in the PAGE Study. Diabetologia. 2022;65(3):477-89.
54. Magi R, Horikoshi M, Sofer T, Mahajan A, Kitajima H, Franceschini N, et al. Trans-ethnic meta-regression of genome-wide association studies accounting for ancestry increases power for discovery and improves fine-mapping resolution. Hum Mol Genet. 2017;26(18):3639-50.
55. Fernandez-Rhodes L, Malinowski JR, Wang Y, Tao R, Pankratz N, Jeff JM, et al. The genetic underpinnings of variation in ages at menarche and natural menopause among women from the multi-ethnic Population Architecture using Genomics and Epidemiology (PAGE) Study: A trans-ethnic meta-analysis. PLoS One. 2018;13(7):e0200486.
56. Jo Hodonsky C, Schurmann C, Schick UM, Kocarnik J, Tao R, van Rooij FJ, et al. Generalization and fine mapping of red blood cell trait genetic associations to multi-ethnic populations: The PAGE Study. Am J Hematol. 2018.
57. Wojcik GL, Graff M, Nishimura KK, Tao R, Haessler J, Gignoux CR, et al. Genetic analyses of diverse populations improves discovery for complex traits. Nature. 2019;570(7762):514-8.
58. Scannell Bryan M, Sofer T, Mossavar-Rahmani Y, Thyagarajan B, Zeng D, Daviglus ML, et al. Mendelian randomization of inorganic arsenic metabolism as a risk factor for hypertension- and diabetes-related traits among adults in the Hispanic Community Health Study/Study of Latinos (HCHS/SOL) cohort. Int J Epidemiol. 2019;48(3):876-86.
59. Scannell Bryan M, Sofer T, Afshar M, Mossavar-Rahmani Y, Hosgood HD, Punjabi NM, et al. Mendelian randomization analysis of arsenic metabolism and pulmonary function within the Hispanic Community Health Study/Study of Latinos. Sci Rep. 2021;11(1):13470.
60. Lee Y, Chen H, Chen W, Qi Q, Afshar M, Cai J, et al. Metabolomic Associations of Asthma in the Hispanic Community Health Study/Study of Latinos. Metabolites. 2022;12(4).
61. Granot-Hershkovitz E, He S, Bressler J, Yu B, Tarraf W, Rebholz CM, et al. Plasma metabolites associated with cognitive function across race/ethnicities affirming the importance of healthy nutrition. Alzheimers Dement. 2023;19(4):1331-42.
62. Noordam R, Sitlani CM, Avery CL, Stewart JD, Gogarten SM, Wiggins KL, et al. A genome-wide interaction analysis of tricyclic/tetracyclic antidepressants and RR and QT intervals: a pharmacogenomics study from the Cohorts for Heart and Aging Research in Genomic Epidemiology (CHARGE) consortium. J Med Genet. 2017;54(5):313-23.

63. Preudhomme LK, Gellman MD, Franceschini N, Perreira KM, Fernandez-Rhodes LE, Gallo LC, et al. Genetic and stress influences on the prevalence of hypertension among hispanics/latinos in the hispanic community health study/study of latinos (HCHS/SOL). Blood Press. 2022;31(1):155-63.
64. Dunn EC, Wiste A, Radmanesh F, Almli LM, Gogarten SM, Sofer T, et al. Genome-Wide Association Study (Gwas) and Genome-Wide by Environment Interaction Study (Gweis) of Depressive Symptoms in African American and Hispanic/Latina Women. Depress Anxiety. 2016;33(4):265-80.
65. Luo K, Chen GC, Zhang Y, Moon JY, Xing J, Peters BA, et al. Variant of the lactase LCT gene explains association between milk intake and incident type 2 diabetes. Nat Metab. 2024;6(1):169-86.
66. Reynolds KM, Horimoto ARVR, Lin BM, Zhang Y, Kurniansyah N, Yu B, et al. Ancestry-driven metabolite variation provides insights into disease states in admixed populations. Genome Med. 2023;15(1):52.
67. Qi Q, Li J, Yu B, Moon JY, Chai JC, Merino J, et al. Host and gut microbial tryptophan metabolism and type 2 diabetes: an integrative analysis of host genetics, diet, gut microbiome and circulating metabolites in cohort studies. Gut. 2022;71(6):1095-105.
68. Granot-Hershkovitz E, Spitzer B, Yang Y, Tarraf W, Yu B, Boerwinkle E, et al. Genetic loci of beta-aminoisobutyric acid are associated with aging-related mild cognitive impairment. Transl Psychiatry. 2023;13(1):140.
69. Kurniansyah N, Wallace DA, Zhang Y, Yu B, Cade B, Wang H, et al. An integrated multi-omics analysis of sleep-disordered breathing traits implicates P2XR4 purinergic signaling. Commun Biol. 2023;6(1):125.
70. Han X, Lains I, Li J, Chen Y, Yu B, Qi Q, et al. Integrating genetics and metabolomics from multi-ethnic and multi-fluid data reveals putative mechanisms for age-related macular degeneration. Cell Rep Med. 2023;4(7):101085.
