# Supplementary material for: Advancements in genetic research by the Hispanic Community Health Study/Study of Latinos: A 10-year retrospective review
Source: HGG Adv. 2024 Oct 29;6(1):100376. doi: 10.1016/j.xhgg.2024.100376 (PMC11754138; doi:10.1016/j.xhgg.2024.100376)
Supplement: Document S2. Article plus supplemental information [file mmc2.pdf]

HGG Advances

REVIEW

Advancements in genetic research by the Hispanic Community Health Study/Study of Latinos: A 10-year retrospective review

Hridya Rao,[1,12] Margaret C. Weiss,[2,12] Jee Young Moon,[3] Krista M. Perreira,[4] Martha L. Daviglus,[5] Robert Kaplan,[3,6] Kari E. North,[7] Maria Argos,[2,8] Lindsay Fernández-Rhodes,[1] and Tamar Sofer[9,10,11,*]

The Hispanic Community Health Study/Study of Latinos (HCHS/SOL) is a multicenter, longitudinal cohort study designed to evaluate environmental, lifestyle, and genetic risk factors as they relate to cardiometabolic and other chronic diseases among Hispanic/Latino populations in the United States. Since the study's inception in 2008, as a result of the study's robust genetic measures, HCHS/SOL has facilitated major contributions to the field of genetic research. This 10-year retrospective review highlights the major findings for genotype-phenotype relationships and advancements in statistical methods owing to the HCHS/SOL. Furthermore, we discuss the ethical and societal challenges of genetic research, especially among Hispanic/Latino adults in the United States. Continued genetic research, ancillary study expansion, and consortia collaboration through HCHS/SOL will further drive knowledge and advancements in human genetics research.

Introduction

Despite being one of the fastest-growing racial/ethnic minority populations in the United States, Hispanic/Latino individuals are underrepresented in biomedical research.[1,2] The Hispanic Community Health Study/Study of Latinos (HCHS/SOL) is a longitudinal, multi-site cohort study that aims to study the health of Hispanic/Latino adults living in the United States. Launched in 2008, 16,415 adult participants were enrolled in HCHS/SOL to characterize and contextualize chronic disease and prospectively examine morbidity and mortality among diverse Hispanic/Latino adults.[3] Community sites recruited participants of diverse Hispanic/Latino backgrounds living in Chicago, IL; the Bronx, NY; Miami, FL; and San Diego, CA.

The implementation of HCHS/SOL substantially extended participant diversity in NIH-funded epidemiologic and genetic studies. In addition to assessing various clinical, demographic, and survey data, genetic measures have been generated from HCHS/SOL participants. Initially, approximately 13,000 consenting participants were genotyped,[4] and recently this number has increased to over 14,000 participants (Figure 1). Figure 2 characterizes HCHS/SOL participants with genetic data. Hispanic/Latino individuals are significantly underrepresented in genetic research, representing 0.34% of participants in genetic studies compared with 19% of the overall adult US population.[5] Since the inception of HCHS/SOL, the study investigators have published over 500 papers leveraging this unique resource, with more than 75 papers providing vital steps toward understanding the complex genetics of cardiovascular and cognitive health among individuals who self-identify as Hispanic/Latino. The overarching goal of this review was to summarize the contributions that HCHS/SOL has made to genetic research since its inception as a landmark population-based study of Hispanic/Latino individuals. Thus, the review is organized by sections covering general and specific areas of genetic epidemiology research and highlights findings from HCHS/SOL in these related areas. Box 1 lays out population descriptors used in this review.

Epidemiologic genetic research

Despite the rapid developments in human genetic research over the past few decades, the absence of adequate representation of the Hispanic/Latino population in genetic studies has been a significant limitation.[6] Since the first genome-wide association study (GWAS) conducted for age-related macular degeneration in 2005, the use of GWAS has soared with screening of over 200,000 genetic variants for over 3,000 traits.[7] Since then, the majority of GWASs have reported variants that were mapped and identified disease susceptibility in European ancestry populations, while non-European continental genetic ancestries have been studied less. For example, African American, South Asian,

[1]Department of Biobehavioral Health, Pennsylvania State University, University Park, PA, USA; [2]Department of Epidemiology and Biostatistics, School of Public Health, University of Illinois Chicago, Chicago, IL, USA; [3]Department of Epidemiology and Population Health, Albert Einstein College of Medicine, Bronx, NY, USA; [4]Department of Social Medicine, University of North Carolina School of Medicine, Chapel Hill, NC, USA; [5]Institute for Minority Health Research, University of Illinois at Chicago, Chicago, IL, USA; [6]Division of Public Health Sciences, Fred Hutchinson Cancer Research Center, Seattle, WA, USA; [7]Department of Epidemiology, University of North Carolina, Chapel Hill, NC, USA; [8]Department of Environmental Health, School of Public Health, Boston University, Boston, MA, USA; [9]Cardiovascular Institute, Beth Israel Deaconess Medical Center, Harvard Medical School, Boston, MA, USA; [10]Department of Biostatistics, Harvard T.H. Chan School of Public Health, Boston, MA, USA; [11]Department of Medicine, Brigham and Women's Hospital, Boston, MA, USA
[12]Co-first authors
*Correspondence: tsofer@bidmc.harvard.edu
https://doi.org/10.1016/j.xhgg.2024.100376.
© 2024 The Authors. Published by Elsevier Inc. on behalf of American Society of Human Genetics.
This is an open access article under the CC BY license (http://creativecommons.org/licenses/by/4.0/).

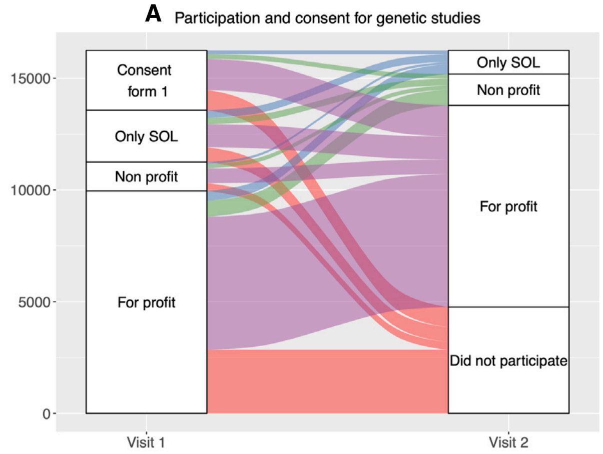

B Genetic data participants

Genotyping effort	Year	Participants	Sample size
OLa: Illumina Omni2.5M + ~150k custom content	2013	Visit 1 participants using IC form 2 and consenting for either only SOL, non profit, or for profit categories	12,803
PAGE II: MEGA array	2017	Visit 1 participants using IC form 2 and consenting for either non profit, or for profit categories	11,864
TOPMed + CCDG	2018-2024 (in stages, freezes 6-12)	Visit 1 participants using IC form 2 and visit 2 participants, consenting for either non profit, or for profit categories.	13,051

Figure 1. Availability of genetic data of HCHS/SOL participants
(A) Patterns of consent for participation in genetic studies in HCHS/SOL Visits 1 and 2.
(B) Genotyping efforts in HCHS/SOL. Initially, 12,803 individuals who were genotyped via the OLa project. Later, individuals were genotyped via the PAGE study together with individuals from other studies. Both OLa and PAGE genotyped data were imputed to the 1000 Genomes phase 3 reference panel and later to TOPMed reference panel. Finally, most of the genotyped individuals and additional individuals were sequenced (WGS) via the NHLBI's TOPMed and the NHGRI's CCDG projects. TOPMed and CCDG genotyped and performed joint allele calling of participants from many parent studies. As of 2024, there are 14,126 HCHS/SOL individuals with genome-wide genetic data across the three levels of informed consent: allowing genetic data to be used only by HCHS/SOL investigators or their collaborators ("Only SOL"), allowing genetic data to be used only by researchers from nonprofit organizations ("Non profit"), or allowing genetic data to be used also by researchers from for-profit organizations ("For profit"). The first 2,694 Visit 1 participants who consented via the first version of the informed consent form ("Consent form 1") were not genotyped initially. CCDG, Centers for Common Disease Genomics; IC, informed consent; NHGRI, National Human Genome Research Institute; NHLBI, National Heart, Lung, and Blood Institute; OLa, omics in Latinos; PAGE, Population Architecture using Genomics; SOL, (Hispanic Community Health Study) Study of Latinos; TOPMed, *trans*-omics in precision medicine.

and Hispanic/Latino individuals, representing non-European or admixed patterns of genetic ancestries, are still underrepresented in genetic research, but cohorts like HCHS/SOL have made significant contributions to begin to fill this gap.[8] As visualized in Figure 3, the Hispanic/Latino population include individuals who are admixed with primarily European, Amerindian, and African ancestries, but this admixture is not homogeneous by region and differs by specific countries of birth.[4] Lack of ethnic and ancestral diversity in genetic research not only limits understanding of the genome's impact on disease but can also contribute to health disparities due to limited translation of the research to precision medicine in underrepresented populations. Racial/ethnic groups with admixed ancestry are still underrepresented. To address these challenges, Figure 4 depicts the numerous genetic studies that HCHS/SOL has published since 2014 and the diversity of the study types. Figure 5 depicts the contributions of HCHS/SOL genetic research to health outcomes research with further detail of the specific studies cited in Table S1. The following sections of this review will summarize HCHS/SOL contributions and findings across several fields of human genetics research.

Genotyping and imputation of HCHS/SOL participants

Participants were asked to fast overnight before their clinic visit. Blood was extracted from participants using a trained phlebotomist to EDTA tubes and was refrigerated and shipped on the same day at 4°C. DNA was extracted from the packed cells of EDTA blood by the HCHS/SOL central laboratory at the University of Minnesota. As described in Figure 1, HCHS/SOL participants were genotyped via three separate efforts. Participants in these genotyping projects differed somewhat based on the individual's level of consent for genetic data sharing, as well as, only for visit 1 participants, the informed consent form used. Initial genotyping was performed via the omics in Latinos (OLa) project via an Illumina array that was customized to HCHS/SOL: array 15041502 B3, which consisted of the Illumina Omni 2.5M array (HumanOmni2.5-8v1-1) and an additional ~150,000 selected SNPs; these SNPs were enriched for ancestry-informative markers and SNPs common in Amerindian ancestries.[4] These genotyping data were initially imputed by the HCHS/SOL Genetic Analysis Center, which existed until 2017. The first imputation panel was the 1000 genome[9] freeze 1, and later 1000 genome freeze 3 was used. Most participants genotyped by OLa were genotyped a few years later via the Population Architecture using Genomics and Epidemiology (PAGE) consortium. PAGE aggregated individual-level data from multiple studies, including HCHS/SOL, and genotyped individuals using Illumina's multiethnic genotyping array (MEGA) and unified protocols. Notably, PAGE investigators developed this array in partnership with Illumina.[10] PAGE initially imputed its participants to the 1000 genome freeze 3 reference panel.

Background group	Age plot	N	Female (%)	Age		Income categories (%)			Education			US born (%)
				Mean, *sd*	Min,max	<$30,000	≥$30,000	*Missing*	<HS	HS	>HS	
Dominican	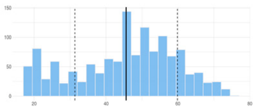	1316	65.7%	45.6, *14.3*	18-75	70.1%	23.6%	6.3%	42.2%	20.8%	36.9%	8.4%
Central American	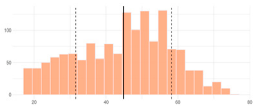	1484	60.2%	44.9, *13.3*	18-75	68.4%	23.3%	8.3%	41.4%	22.7%	35.8%	4.6%
Cuban	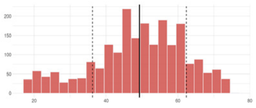	2158	52.5%	49.3, *13.1*	18-75	67.8%	22.4%	9.7%	23.0%	29.3%	47.6%	5.1%
Mexican	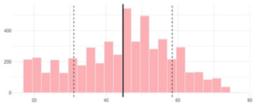	5345	61.4%	44.7, *13.7*	18-76	60.9%	35.1%	3.9%	43.3%	26.2%	30.4%	17.3%
Puerto Rican	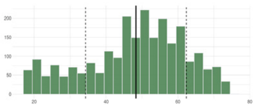	2406	58.4%	48.3, *14.0*	18-75	63.6%	31.5%	4.9%	39.0%	26.8%	34.2%	41.6%
South American	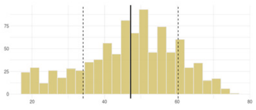	905	58.6%	47.2, *13.1*	18-76	64.9%	31.6%	3.5%	24.0%	25.6%	50.4%	4.4%
More than one/Other heritage	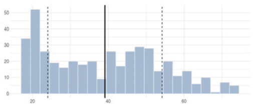	428	54.2%	48.3, *14.0*	18-75	54.4%	38.1%	7.5%	25.7%	20.8%	53.5%	57.0%

Figure 2. Characteristics of HCHS/SOL participants who consented to genetic analyses ($N = 14{,}042$)
Characteristics are based on the baseline visit are stratified by self-reported Hispanic/Latino background. The figure shows age distributions and lists the sample size, percentage of female individuals, mean, standard deviation, and range of age, percentage of participants with income less than or greater than or equal to 30,000 USD per household per year, percentages of participant by education categories (no high school diploma or GED, at most a high school diploma or GED, greater than high school [or GED] education), and percentage of participants who were born in the 50 US states/DC (US born). Sex was assigned in visit 1 and is presumed to be the sex assigned at birth.

Finally, many of HCHS/SOL participants were sequenced by whole-genome sequencing (WGS), supported by two initiatives: the National Heart Lung and Blood Institute's Trans-Omics in Precision Medicine (TOPMed) and the National Human Genome Research Institute's Centers for Common Disease Genetics (CCDG) projects. These two efforts collated samples from many existing US and sometimes, international, studies, performed WGS via multiple sequencing centers while implementing uniform protocols, followed by joint allele calling. Due to the large number of samples, these sequencing studies were performed in multiple steps, where every joint TOPMed-CCDG "freeze" included an increased number of individuals compared with the previous one. The first imputation reference panel that used the TOPMed-CCDG data was based on their freeze 5 dataset. Once it became available, HCHS/SOL chip genotyping data were imputed by investigators to the TOPMed reference panel.[11]

Methodology for addressing population stratification

There are several limitations to applying standard GWAS designs to genetic data from HCHS/SOL. Researchers have begun to develop methods to alleviate these limitations, and the benefits of these methods often extend beyond analysis of HCHS/SOL data. For example, the phenomenon where genetic patterns can be identified as differing between groups of individuals is known as population structure.[12] Population stratification is used to describe the settings in which population structure is correlated with systematic differences in phenotypes, but in a manner that is not necessarily related to the underlying biological mechanisms.[13] Effectively accounting for population structure is a major challenge when conducting genetic association studies using an admixed study population.[14] While in the early days of GWAS population

Box 1. Population descriptors used in this review

Genetic ancestry	Race/ethnicity and Hispanic/Latino background
**Genetic ancestry:** A term to refer to one's genetic descent. In this review, we mostly use the term genetic ancestry to refer to discrete, pre-defined, ancestral populations from which segments of an individual's DNA were inherited, but genetic ancestry is also quantified by continuous measures such as genetic principal components. Discrete characterization of genetic ancestry depends on the reference population used and timescale investigated.<br>**Continental genetic ancestries:** The description of genetic ancestries according to genetic patterns observed over populations represented by individuals grouped at the continental level, e.g., European, African, East Asian, South Asian, etc.<br>**Admixture:** The mixing or blending of populations that have been isolated. The ancestral groups are assumed to have been differentiated long before individuals began to mix and have distinct patterns of allele frequencies and linkage disequilibrium. Hispanic/Latinos are admixed with, primarily, European, Amerindian, and African genetic ancestries.<br>**Amerindian ancestry:** A descriptor that refers to the genetic patterns characterizing indigenous peoples of the Americas, who are also commonly referred to as Native American or American Indian. Many HCHS/SOL papers have used the term Amerindian ancestry, but other terms like Amerindigenous have also been used.<br>**Latin American or Hispanic ancestry:** Terms that refer to the genetic patterns of people living in Latin America, or who are from Latin America. This is distinguished from African, Amerindian, and European genetic ancestries, which refer to the ancient, parental, ancestral populations.	**Ethnicity:** The quality of belonging to a particular socially defined group or national identity that is made of people who share a common ancestral descent or cultural background. In the United States, the Office of Management and Budget defines two primary ethnic categories—Hispanic or Latino, or not Hispanic or Latino.<br>**Race/ethnicity:** The cross-categorization or grouping based on the combination of one's "race" and "ethnicity." Often self-reported in research by participants based on their perceived belonging to an ancestral, socio-cultural, or ethnic group. In US-based studies, individuals are typically grouped into non-Hispanic White, Black, etc., and Hispanic or Latino (regardless of race).<br>**Hispanic/Latino**: A descriptor relating to individuals from Spain or Spanish-speaking countries in Latin America. This amalgamated term is typically used in HCHS/SOL publications to refer to Hispanic or Latino individuals. The Office of Management and Budget in the United States defines Hispanic or Latino origin to include Mexican, Puerto Rican, Cuban, Spanish, or other Spanish-speaking country in Central or South America. Hispanic/Latino can further refer to any individual living in the United States who identifies themself as having background or heritage from any of these Spanish-speaking groups. Individuals with Brazilian heritage may also identify as Latinos. HCHS/SOL does not include individuals from Spain.<br>**Hispanic/Latino background:** A descriptor used in HCHS/SOL of one's Hispanic/Latino heritage, which can be traced back to former Spanish or Portuguese holdings in North America and the Caribbean including Mexico, Cuba, the Dominican Republic, and Puerto Rico, as well as most countries of Central and South America.

stratification was studied in the context of case-control studies,[15] it was later acknowledged that population structure leads to population stratification in analysis with quantitative traits.[16] HCHS/SOL is an admixed population comprising individuals from varied Hispanic/Latino backgrounds that demonstrate genetic substructure. HCHS/SOL investigators also demonstrated population stratification phenomenon at the trait variance level: differences in trait variance among groups, coupled with differences in allele frequencies, led to inflated or deflated standard error estimation. While this phenomenon was also described earlier[17] with the proposal of modeling trait variance as a function of genetic principal components (PCs), HCHS/SOL investigators demonstrated the use of group-specific residual and genetic variances to reduce such population stratification effects.[4,18,19] Given the relative simplicity and computational efficiency of modeling of heterogeneous residual variance by group, this was implemented in the GENESIS R package.[20]

Another example of a population stratification challenge to association analyses that came to light, with a proposed solution based on work in HCHS/SOL, is in the analysis of binary traits. While the original reports of population stratification were in the space of case-control studies, they focused on population structure differences between cases and controls, and the problem was (relatively) effectively addressed by PC adjustment or usage of a genetic relationship matrix in linear mixed models. Analyses in HCHS/SOL, due to aggregation of individual-level data of participants from different Hispanic/Latino background groups that demonstrate population substructure (e.g., Mexican, Dominican, etc.), revealed that the use of linear mixed model resulted in improper control of type 1 error when the binary outcome has different prevalence between groups. This is because in linear models the mean is unrelated to the variance, while in logistic models, there is a specific mean-variance relationship. The generalized linear mixed model association test (GMMAT) alleviated this analytical roadblock.[21] Finally, another study, in collaboration with HCHS/SOL, developed a *trans*-ethnic meta-regression model and demonstrated that it increases the statistical power of a meta-analysis when allele effects are correlated with genetic ancestry.[22]

Generalization and transferability of associations from other populations to Hispanic/Latino individuals

Because genetic studies have been traditionally dominated by populations of primarily European ancestries, one of the major concerns of genetic research in HCHS/SOL, as well as other studies of individuals of diverse, non-European, genetic background, has been the utilization of

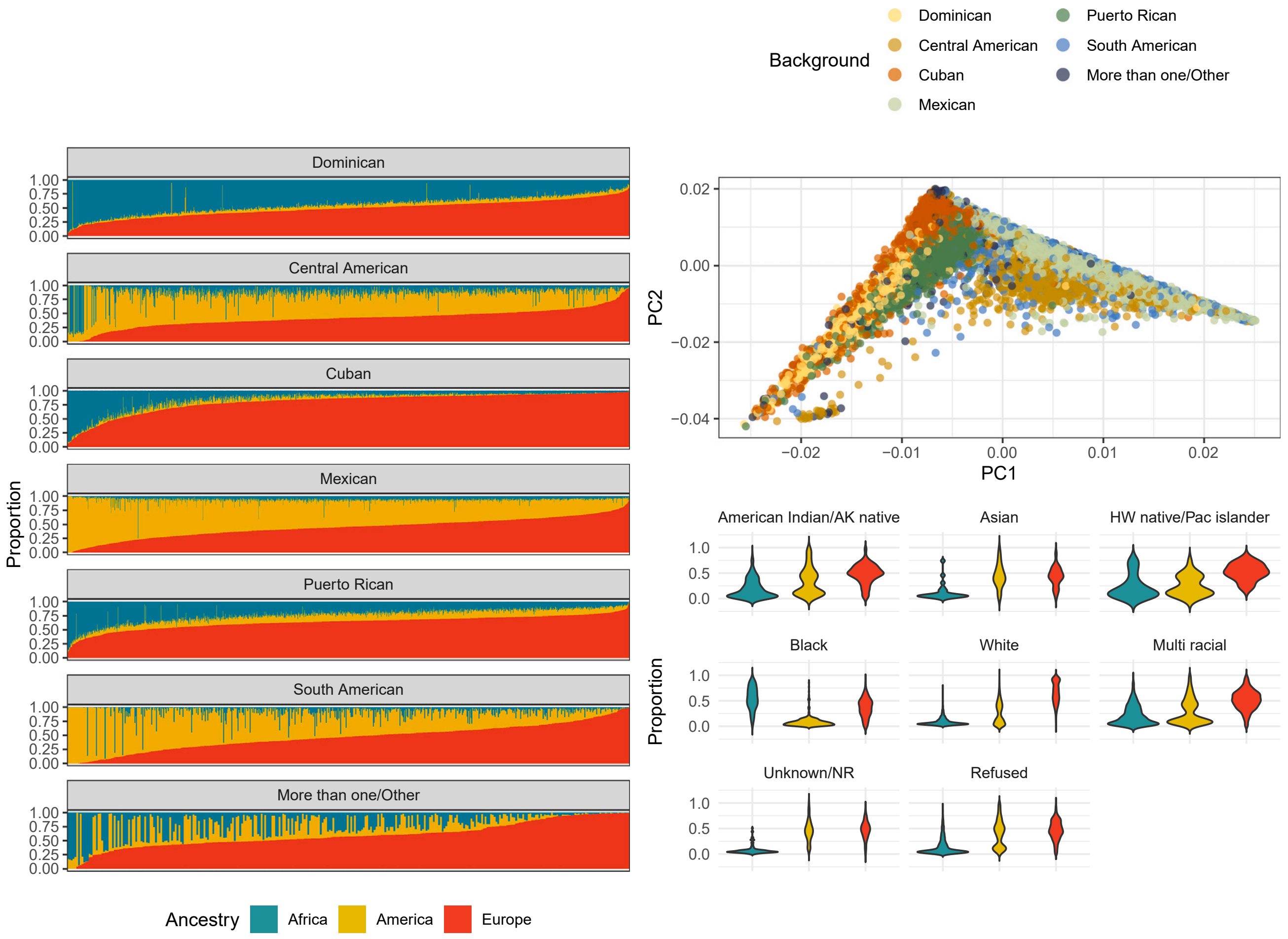

Figure 3. Self-reported Hispanic/Latino background, race, and ancestry in HCHS/SOL
The figure is limited to 10,471 individuals with non-missing self-reported Hispanic/Latino background and race, inferred global genetic ancestry proportions, and who consented for genetic data sharing with either HCHS/SOL investigators or other nonprofit or for-profit researchers. Left: proportions of inferred African, Amerindian, and European continental ancestries for HCHS/SOL participant of each Hispanic/Latino background (Dominican n = 962; Central American n = 1,146; Cuban n = 1,768; Mexican *N* = 3,753; Puerto Rican n = 1,825; South American n = 793; More than one/Other n = 314). This figure demonstrates there are background-specific patterns of global genetic ancestries, which are known to be due to demographic history. Top right: values of the first and second genetic PCs, with points colored by self-reported Hispanic/Latino background. PC1 explains 2.55% and PC2 explains 1.12% of the genetic variance in the dataset.[4] This figure demonstrates that Hispanic/Latino backgrounds tend to cluster together on PC space. Bottom right: distribution of inferred proportions of genetic ancestry by categories of self-reported race (American Indian/AK native n = 405; Asian n = 23; HW native/PC islander n = 25; Black n = 359; White n = 4,055; Multi-racial n = 1,790; Unknown/NR n = 112, Refused n = 3,702). This figure demonstrates that self-reported race is only weakly related to patterns of admixture of ancestral population ancestries in HCHS/SOL Hispanics/Latinos. AK, Alaska; HCHS/SOL, the Hispanic Community Health Study/Study of Latinos; HW, Hawaii; NR, non-response; Pac, Pacific; PC, principal component.

European-derived genetic estimates in populations with different genetic ancestries. A method developed by Sofer et al. in 2017 provided a formal statistical framework for quantifying generalization for genotype-phenotype associations from previously reported studies to a new study, while controlling the false discovery rate of the generalization null hypothesis.[23] This study found that many genetic associations first identified in European populations generalize to Hispanic/Latino populations. However, look-ups of European-derived results in HCHS/SOL GWAS results demonstrated low power for generalization testing. Various manuscripts addressed this by performing association testing of genetics scores utilizing variants that have not been generalized in single-variant tests. These are weighted (with weights being the estimated effect sizes from GWASs) or unweighted sums of trait-associated alleles. This was done, for example, in studies of diabetes and anthropometric traits genetics,[24,25] showing that many associations found in European populations likely also exist in Hispanic/Latino individuals.

Unique study design contributing to assessment of genetic and environmental contributions to phenotypes

HCHS/SOL individuals were recruited to the study in a multi-step sampling approach, where ultimately some

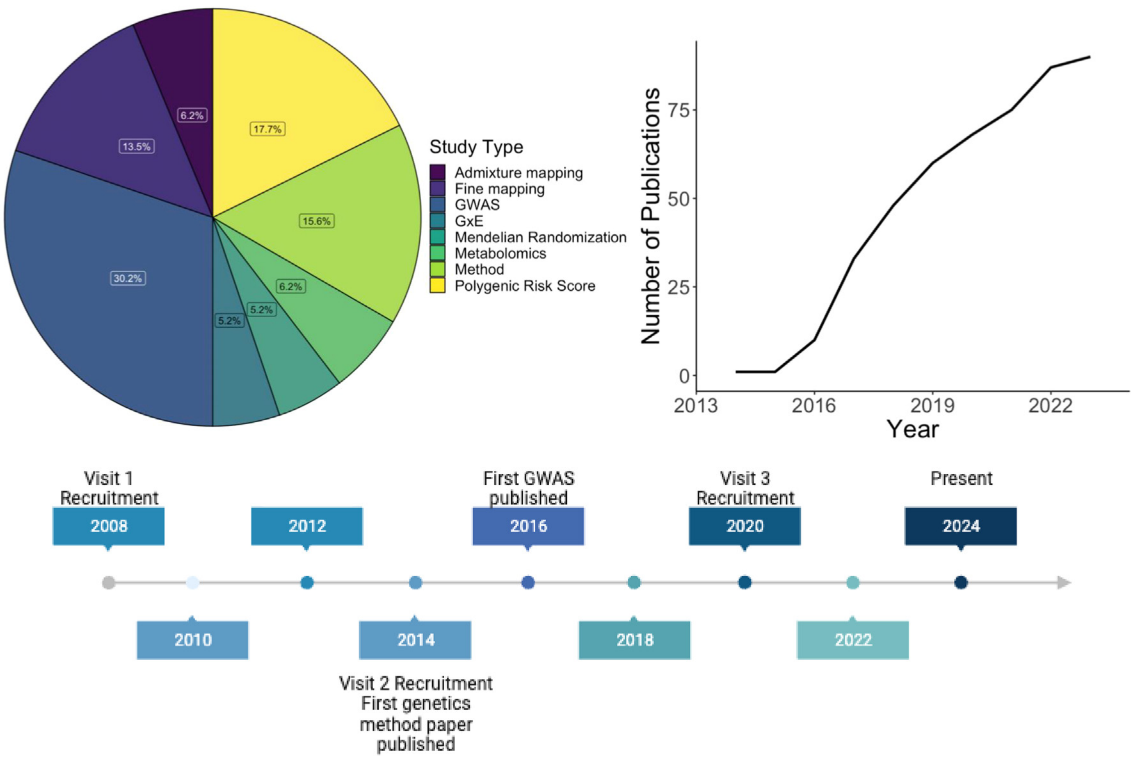

Figure 4. Cumulative number and type of human genetic studies published from HCHS/SOL since 2014

individuals were sampled from the same household.[26] This allowed for estimation and comparison of both genetic and "household" contribution to trait variability and relationship between phenotypes. Household contribution refers to a collection of environmental and behavioral factors that are common for individuals living in the same house. For example, Sofer (2017) described the use of Haseman-Elston regression to estimate both heritability and variance explained by household,[27] and showed that household environment substantially contributes to height (10% estimated variance explained, compared with 58% heritability), depression score (6% estimated variance explained compared to 4% heritability), and it is responsible for an estimated ~22% variance explained of periodontitis (compared with statistically insignificant estimated heritability <3%). Similarly, the methodology was extended for estimation of correlations, where both genetic and household correlations (i.e., the correlation between traits due to additive genetic and household effects, respectively) were estimated.[28] This analysis demonstrated, for example, that the relationship between diabetes and lipid phenotypic domains are mostly due to genetics and not due to shared household environment, the association between diabetes and blood pressure domains are mostly due to shared household rather than genetics, and associations between anthropometrics and sleep domains are due to both genetics and household environments. With currently ongoing assessment of genetic profiles of SOL Youth participants,[29] who are children of HCHS/SOL adults, we expect more HCHS/SOL research to develop into assessments of "nature versus nurture."

Inference relating to the HCHS/SOL population history and ancestral populations

Browning and colleagues in 2018 utilized HCHS/SOL to estimate ancestry-specific population sizes based on local ancestry and identity by descent analyses,[30] finding that the pre-admixture effective population sizes of the HCHS/SOL ancestral populations varied by group of different Hispanic/Latino background. This implies that Hispanic/Latino populations of different backgrounds were drawn from different subpopulations of the ancestral populations. Local ancestry inference was later utilized for multiple admixture mapping analyses: analyses that estimate association between counts of genetic ancestry localized to genetic regions, with a trait, with associations relying on differences in allele frequencies between the ancestral populations used.[31–33] Grinde et al. (2019) developed a method to quantify the multiple testing burden in admixture mapping.[34] Zhang et al. developed the ancestry-specific allele frequency estimation (ASAFE) method[35] to estimate ancestry-specific allele frequencies in 3-way admixed populations, and this method was later improved by Granot-Hershkovitz et al. with the allele frequency estimation in admixed populations (AFA) method,[36] which is computationally efficient enough that it was applied

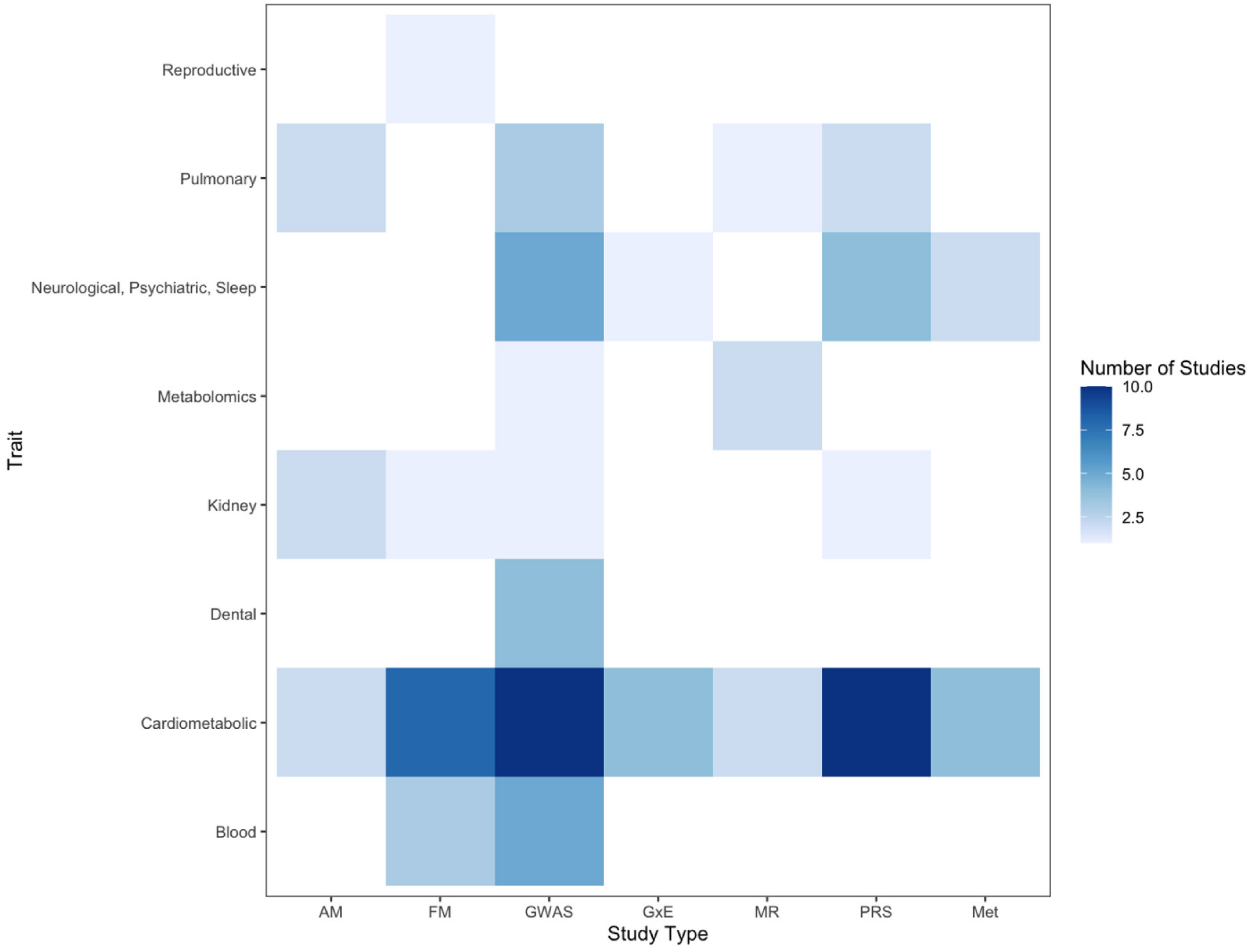

Figure 5. Heatmap depicting genetic epidemiological study types and studied health outcomes conducted using HCHS/SOL data
White tiles correspond to no publications in the category. AM, admixture mapping; FM, fine mapping; GWAS, genome-wide association study; GxE, Gene-by-environment interaction study; MR, Mendelian randomization; PRS, Polygenic Risk Score; Met, Metabolomics.

genome-wide. Knowing the ancestry-specific allele frequencies of genetic variants is useful for replicating genetic associations discovered in an admixed population and identifying high-risk groups that may be more affected by a risk locus. For example, identification of variants in GWASs that had large Amerindian, rather than European or African, ancestry-specific allele frequency, guided the collaboration with Pima Indian population to replicate variant associations with Albuminuria[31] and with blood pressure phenotypes.[32]

Genome-wide association study

HCHS/SOL-specific GWASs were performed to validate and identify variants for several chronic diseases, including cardiovascular disease, type 2 diabetes (T2D), dental disease, anxiety, depression, and sleep apnea.[25,37–40] These studies helped discover genetic loci that were more common in Amerindian and African ancestries compared with European ancestries. Moreover, these GWASs showed that many findings from other race/ethnicity and ancestrally diverse cohorts are generalized to HCHS/SOL. The extensive impact of genotype-phenotype associations using GWASs conducted with HCHS/SOL is summarized in Table 1.

Admixture mapping

HCHS/SOL has been utilized to conduct admixture mapping studies to identify genomic regions associated with phenotypic traits. Several phenotypes have been assessed, including cardiovascular traits like blood pressure and lipids, pulmonary traits including lung function and sleep apnea, and kidney traits including albuminuria and chronic kidney disease.[31,33,52] Admixture mapping relies on patterns of admixture localized to genomic regions to identify specific regions where trait-associated variants reside. Such associations can be detected when the causal variant allele frequencies differ between the ancestral populations. Admixture mapping is performed by testing the association of counts of local ancestry alleles (i.e., counts of variant alleles or chromosomal segments covering a

Table 1. Summary of GWASs

Phenotype/Outcome	Primary findings	Genes	Replication	Reference
**HCHS/SOL-only studies**				
Dental caries	GWAS of DMFS and DMFT. Study estimated heritability of dental caries between 20% and 53%. SNPs associated with several biological processes including periodontal healing and tooth development were identified.	Variant upstream of *NAMPT* was associated with dental carries significantly ($p < 5 \times 10^{-8}$). Association with a variant downstream of *BMP7* was also found.	Replication in an independent sample was not sought.	Morrison et al., 2016[41]
Chronic periodontitis	GWAS of CP identified 1 novel genome-wide significant locus (1q42.2) at the *TSNAX-DISC1* noncoding RNA and 4 suggestively significant loci.	Strongest association of CP in HL participants was in the *TSNAX-DISC1* noncoding RNA region. Associations were also found with *ASH1L, NELL1, IRX1; LINC01017, LINC01019, LOC645157,* and *RNF144B* genes.	Replication of the GWAS findings was performed in cohorts with EUR and AFR individuals. No replication in EUR; however, study replicated the association at *TSNAX-DISC1* locus in AFR sample.	Sanders et al.[38]
Platelet count	GWAS of platelet count revealed 5 novel associations that were replicated. GWAS identified 9 genome-wide significant and 3 suggestively significant loci. Seven out of 10 SNPs previously identified in AFR were generalized to HL. Half of 49 EUR SNPs and 4 EAS SNPs were also generalized. Strongest association was with Amerindian-specific variant - rs117672662.	Strongest associations were in/near genes such as, *ACTN1, ETV7, GABBR1-MOG, MEF2C,* and *ZBTB9-BAK1*.	Four out of 5 SNPs were replicated	Schick et al., 2016[42]
RBC traits	GWAS of RBC traits, such as RBC count, distribution width and indices were carried out to reveal 7 novel signals.	Novel, genome-wide significant signals were in/near the *PROX1, RBFOX3, SLC12A2, PSMB5, MCTP2,* and *IDO2* genes.	Three out of 7 signals were successfully replicated (at *PROX1, SLC12A2,* and *PSMB5* genes).	Hodonsky et al., 2017[43]

(*Continued on next page*)

Table 1. *Continued*

Phenotype/Outcome	Primary findings	Genes	Replication	Reference
WBC count	Study performed GWAS of WBC counts in HCHS/SOL and found rare germ-line variants associated with MON count. They also identified loci associated with or regulating hematopoietic transcription factors. Minor allele of *CEBPE* was associated with BAS, previously known to increase ALL in HL populations. They also generalized several previously identified loci.	Rare germ-line variant in *FLT3* was associated with monocyte count. Several variants in genes *CEBPE-SLC7A7, CEBPA, CEBPE,* and *CRBN-TRNT1* were linked to BAS.	Ten associations were tested in a validation sample, of which 4 were replicated.	Jain et al., 2017[44]
Iron	Study conducted the first GWAS of TIBC, transferrin saturation, and ferritin and identified a novel variant and this effect strengthened when excluding iron deficient individuals. Study also generalized 10 of 16 previously identified iron SNPs. They identified a link between iron traits and lower HbA1c levels.	Study's novel signal was near the *PPP1R3B* gene known for protein phosphatase activity. Study generalized previous associations in/near genes such as, *TMPRSS6, MYRF, MYRF,* and *GAB3*.	Top signal was replicated in EUR meta-analysis. *TIBC* association was found to be opposite in direction and non-significant in one of their replication cohorts (JHS).	Raffield et al., 2017[45]
Blood pressure traits	GWAS of SBP and DBP revealed 7 genome-wide significant SNPs and 1 suggestively significant SNP previously unreported. Study generalized loci previously identified in EUR, EAS, and AFR populations.	Associations between genes such as *FGF5, SCGN, NRG3, and SLC5A8* and BP traits were found.	Replication was sought in multi-population cohorts and 2 loci were marginally replicated.	Sofer et al., 2017[46]
Cognitive function	Four novel loci associated with cognitive function measures such as Brief Spanish English Verbal Learning Test, Word Fluency Test were identified.	Identified loci have been linked to genes associated with brain tissue and neurological diseases, such as *UBE2K, FRMD4B,* the *HLA* gene complex.	Though the SNPs were not replicated in their independent validation sample, they were directionally consistent.	Jian et al.[47]

(*Continued on next page*)

Table 1. *Continued*

Phenotype/Outcome	Primary findings	Genes	Replication	Reference
HbA1c	Twelve genome-wide significant associations with HbA1c were found, of which 6 were novel. Four signals were associated at a nominal significance threshold ($p < 0.05$).	Novel signals were found in *HBB, HBM, TMC6,* and *G6PD* genes in the discovery GWAS and replicated.	Replication was sought in the HL population and revealed some robust associations.	Moon et al., 2019[48]
Lipid traits	GWAS of lipid traits such as LDL, HDL, TCL, and TGL revealed 14, 16, 17, and 10 genome-wide significant independent loci respectively. Conditional analyses yielded 8 novel signals, 6 of which were potentially novel primary signals.	Associations between HDL and *APOA5/A1* and *DAGLB* regions were identified. Potentially novel primary signals associated with HDL were in *SYNE1* and *AUTS2*. A signal in *DNAL1* was associated with LDL, and a signal in *SMOC2* was associated with TGL. *CD86* and *DNAH5* harbored potentially novel primary signals for TCL.	Replication was carried out in cohorts with Hispanic/Latino individuals, and also in European and African ancestry cohorts to generalize some rare variants.	Graff et al., 2017[49]
Central adiposity	GWAS of WHR, WC, HIP adjusting for BMI were conducted. Study identified 16 variants for WHR adjusted by BMI (WHRadjBMI), 22 for WCadjBMI, and 28 for HIPadjBMI at a suggestive significance level of $p < 1 \times 10^{-6}$. They also generalized several loci previously associated with these outcomes.	Genes such as *CDK5RAP2, SLC22A18AS* were revealed to be harboring SNPs associated with WHRadjBMI. *FADS2* and *IRF2BPL* were found to be associated with WCadjBMI and HIPadjBMI, respectively. These were novel loci that were replicated in an independent sample.	Validation cohorts included HL, AFR, and EUR participants and study replicated 4 novel loci in them.	Justice et al.[24]
ABI and PAD	GWAS of ABI revealed 2 genome-wide significant associations in Puerto Rican and Caribbean background groups. Study was not able to generalize previously identified EUR SNPs for ABI or PAD in HL.	The lead SNPs at the genome-wide significant loci were in *COMMD10* (association found in Puerto Rican individuals) and in *DMD* (association significant in Caribbean populations).	Replication of some SNPs, such as rs4466200, in MESA AAs was achieved, albeit not in CHS AAs.	Sofer et al., 2019[50]
Smoking	GWAS of smoking behaviors identified some signals associated with heavy smoking and nondaily smoking in HCHS/SOL participants.	A signal associated with heavy smoking was in the known region of *CHRNA5*. *CHRNA4* was suggestively significant in line with previous findings in non-Hispanic individuals. Two novel signals for nondaily smoking, one downstream of *CCDC85A* and one in the *ENPEP* region were identified at genome-wide significance level.	Replication was sought for nondaily/daily smoking phenotype and the 2 novel signals identified in discovery were not replicated in the validation samples.	Saccone et al., 2018[51]

(*Continued on next page*)

Table 1. *Continued*

Phenotype/Outcome	Primary findings	Genes	Replication	Reference
FEV_1, FEV_1/FVC, airflow limitation, and COPD	Seven genome-wide significant signals were identified, of which 2 were novel for lung function.	SNP in *ZSWIM7* was found to be associated with FEV_1. An intronic variant in *HAL* was associated with FEV_1/FVC, which remained significant after conditioning on previously identified signals from nearby genes. Variants in/near *PDZD2* and *CDRT15P1* were significantly associated with COPD.	Findings were replicated in EAS, HL, and AA.	Burkart et al.[52]
Anxiety	A GWAS of GAD identified a novel SNP nominally associated with GAD adjusting for psychiatric medication use and significantly associated with GAD in the analysis that excluded medication users.	Associated SNP was intronic to thrombospondin 2 gene *THBS2.*	Replication did not support the findings.	Dunn et al., 2017[53]
Depression	Depression was assessed using the 10-item Center for Epidemiological Studies of Depression Scale and 3 phenotypes of depression were used for GWAS. Combined analyses did not reveal any novel or genome-wide significant loci while sex-stratified analyses identified 6 novel loci in males and 1 in females.	Associations with SNPs near genes such as *FBN3, TYW1* were identified with depression.	Replication was performed in 3 independent cohorts consisting of HL individuals, although none of the significant SNPs were replicated.	Dunn et al. 2018[39]
Temporomandibular disorder	A GWAS of TMD revealed one suggestively significant locus which was replicated—2 additional loci were identified but were not replicated. Sex-stratified GWAS found 2 additional genome-wide significant loci in females.	*SGCA* gene was linked to the suggestively significant locus that was replicated. Other genes were *RXP4* was linked to an SNP found to be associated with TMD in females. Discovery GWAS also revealed associations with *DMD* and *SGCA* genes, but this association was not replicated.	Replication was carried out via a meta-analysis of 4 independent cohorts.	Sanders et al.[38]
PR interval	GWAS of PR interval, a crucial measure associated with atrial fibrillation, heart failure, and all-cause mortality, identified a novel genome-wide significant locus at *ID2*. Study generalized 10 previously identified PR loci.	Lead SNP at the strongest signal was at *ID2*.	Replication confirmed study's top signal.	Seyerle et al.[54]

(*Continued on next page*)

Table 1. *Continued*

Phenotype/Outcome	Primary findings	Genes	Replication	Reference
ACR and eGFR	GWAS identified 15 loci for eGFR and 12 for ACR at $p < 1 \times 10^{-7}$. Study also validated 6 SNVs from the Haas et al. study conducted in UKB.	Associations with variants at *AQR* and *HBB* were found for eGFR and ACR was associated with variants in *BCL2L11, ZBTB16,* and *HBB*.	Signal at *AQR* gene was replicated in HL of WHI study – although a common variant in AFR, this variant was not significantly associated with eGFR in WHI AFR samples. Same for *PRNT,* albeit a nominally significant association. Other variants were directionally consistent.	Qian et al., 2020[55]
Metabolome	GWAS of 640 circulating metabolites revealed 46 variant-metabolite pairs ($p < 1.2 \times 10^{-10}$). Several variants were generalized, and half of them were located in genes, including *SLC51A*.	Strongest associations were in/near *NAT8* and *TPRKB*. Novel associations between 12 metabolites and 6 genes (*PCMT1*, *PTER*, *FOLH1*, *TMEM86B*, and *EEF1A2)* were found. Signal upstream of *SLC51A* was colocalized with the gene in several tissues.	Half the metabolites considered in discovery were available for generalization but sample size requirements were not met.	Feofanova et al.[56]
Genetic variation of microbial taxa	GWAS of microbial taxa revealed 31 loci. Study was pivotal in identifying an age-dependent relationship between the LCT gene locus and *Bifidobacterium* abundance. Other loci enriched for genes in intestine and brain were identified.	*LCT* locus, rs182549 ($p = 1.28 \times 10^{-20}$), was significantly associated with *Bifidobacterium.* Other suggestively significant associations included *Ruminococcus torques-FUT2* gene, *Ruminococcus gnavus-FUT2 gene, Allisolnella- PCSK5, RFK, and GCNT1* associations, among others.	Was not sought due to methodological differences in microbial data collection, their annotation, and weak effect sizes from underpowered studies.	Kurilshikov et al., 2021[57]
**Consortia studies**				
Dental caries experience via DMFS/dentures, DFSS, and Nteeth	As part of the GLIDE consortium, authors sought to perform GWAS of dental caries experience. They found 47 conditionally independent loci with at least 1 genome-wide significant variant for DMFS/dentures. Test for heterogeneity in effect sizes were performed using HCHS/SOL data but excluding it in meta-analysis and comparing it with HCHS/SOL-specific results for lead SNPs. No strong evidence of heterogeneity was found.	Genes such as *KRTCAP2, KRTCAP2, C5orf66, FGF10, MC4R*, and 42 others were associated with DMFS/dentures.	No replication was sought.	Shungin et al., 2019[58]

(*Continued on next page*)

Table 1. *Continued*

Phenotype/Outcome	Primary findings	Genes	Replication	Reference
WBC, BAS, EOS, LYM, MON, and NEU, PLT, and MPV	PAGE study data were used to perform GWAS of hematological traits in AA, HL, and EAS populations to leverage diversity, generalize and identify loci. Genome-wide significant loci were found for all 8 traits in ancestry-specific and combined GWAS.	Discovery analyses identified association of LYM and BAS outcomes with *INSIG1*. Study also identified 4 novel variants in previously identified loci. Ancestry-specific analyses showed *MED13L* and *HADHB* are monomorphic in EAS, while *PPP1R16B* was monomorphic in HL and AA populations.	Replication was sought in Blood Cell Consortium and none of the 6 novel signals were replicated, although they were directionally consistent except the *MED13L* variant.	Hu et al., 2021[59]
BP	GWAS of hypertension traits (SBP, DBP, PP, and HTN) revealed 72 variants significant at $p < 1 \times 10^{-6}$ and HCHS/SOL samples were part of the replication analyses.	Genes such as, *TARID/TCF21, FRMD3, LLPH/TMBIM4, ULK4, PLEKHG1, HOXA,* and others were associated with signals identified in this GWAS with AFR ancestry samples only in discovery.	Trans-ethnic replication did not yield any significant loci in HL.	Liang et al., 2017[60]
SBP and DBP	Through genome-wide association meta-analyses of diverse cohorts, the study found 81 genome-wide significant loci from stages 1 and 2. They generalized 56 known BP loci and identified 10 loci with significant interactions with smoking status.	Study found genes associated with vascular structure and function, such as, *CDKN1B, BCAR1-CFDP1, PXDN,* and *EEA1*. Genes such as, *SDCCAG8* and *RPGRIP1L* associated with ciliopathies were identified. They also found genes associated with telomere maintenance and dopaminergic signaling.	Replication was performed as part of the 2-stage analysis. A combined analysis of both stages was also done to compare. Fifteen novel and 40 known loci were replicated.	Sung et al., 2018[61]
Asthma	Study aimed to generalize asthma susceptibility loci to Puerto Rican populations. HCHS/SOL was one of the discovery cohorts. The study was able to generalize one lead SNP in the 17q21 locus, which also harbored other significantly associated SNPs. An EUR variant and North American population variant were also generalized while some others were not, signifying a distinct genetic architecture for asthma in Puerto Rican individuals.	Study was able to generalize the association between asthma and an SNP at *IKZF3* (which was their lead SNP with greatest effects on asthma in Puerto Rican individuals). They further generalized the association with *ZPBP2*, *ORMDL3*, and *GSDMB* from the same LD block. Associations with variants in *IL1RL1, TSLP,* and GSDMB regions but not IL33 were also generalized.	No further replication other than the study's main aim was sought.	Yan et al., 2017[62]
COPD	Whole-genome sequence analysis of lung function and COPD revealed 10 known and 22 distinct genome-wide significant loci.	Genome-wide significant SNPs that were successfully replicated were associated with *FTO, PIAS1,* and *RGN* (2 variants).	Four out of 22 novel loci were replicated in UKB EUR population.	Zhao et al., 2020[63]

(*Continued on next page*)

Table 1. *Continued*

Phenotype/Outcome	Primary findings	Genes	Replication	Reference
HR variability	GWAS of HR variability revealed 7 loci associated with 3 HR variability traits (RMSSD, SDNN, and HR). Study generalized 11 of 21 previously reported HR SNPs to HL.	Identified signals were in/near genes such as *LINC00477, FUT5, PP1L1, C14orf177, NHLH2, OPCML,* and *OPCML*.	Three genome-wide significant HR variability loci successfully replicated in the EUR and AFR samples.	Kerr et al., 2017[64]
Sleep quality	GWAS of PSQI in a multi-ethnic discovery cohort identified 2 novel genome-wide significant loci on chromosomes 2 and 7.	Identified signals have links to genes *NPY* and *MPP6*, and these have established functions related to sleep. Study also tested an ortholog of *MPP6* gene in fruit flies and it resulted in decreased sleep duration.	Study performed replication of GWAS hits in 12 independent cohorts.	Khoury et al., 2021[65]
QT interval	GWAS of individuals from 4 studies including HCHS/SOL yielded 41 genome-wide significant loci associated with QT interval. Of the 13 lead SNPs, two HL SNPs in/near genes *KCNE1* and *SCN5A* were not correlated with previously reported SNPs.	Thirteen genome-wide significant loci had lead SNPs in/near *ATP1B1, NOS1AP, SCN5A, KCNQ1, SETD6, SLC8A1,* among others. The first 4 genes listed above were also genome-wide significant in meta-analyzed conditional analysis.	No replication was sought, however, several SNPs from previous publications were generalized to HL individuals.	Méndez-Giráldez et al., 2017[66]
Ventricular ectopy	Study performed GWAS of SVE and VE in a multi-cohort sample of 43,000 participants from AFR, EUR, HL ancestry. While trans-ethnic analyses didn't reveal novel associations, variants associated with SVE and VE were found in EUR and AFR subpopulations.	Association at an intronic SNP in *FAF1* previously associated with QRS interval duration was found multi-trait analysis in EUR participants, similarly, a variant near *DSC3* was identified among AFR participants.	No replication was sought due to sample unavailability.	Napier et al., 2018[67]
QRS duration	GWAS of QRS duration showed 6 associations, of which 2 were novel. Roughly 75% of previously known loci were generalized.	Novel associations were in/near genes, *MYOCD* and *SYT1*. Associations with known loci at *SCN5A/SCN10A, CDKN1A, VTI1A,* and *HAND1* were also found.	Replication was not sought in this study.	Swenson et al.[37]

AA, African American; ABI, ankle brachial index; ACR, albumin to creatinine ratio; AFR, African ancestry; ALL, acute lymphoblastic leukemia; BAS, basophil; BMI, body mass index; BP, blood pressure; CHS, Cardiovascular Health Study; COPD, chronic pulmonary obstructive disease; CP, chronic periodontitis; DBP, diastolic blood pressure; DMFS, decayed, missing, and filled tooth surfaces; DMFT, decayed, missing and filled teeth; DFSS, sum of decayed and filled tooth surfaces per available tooth surface; EAS, East Asian; eGFR, estimated glomerular filtration rate; EOS, eosinophil; FEV_1, forced expiratory volume; FVC, forced vital capacity; GLIDE, Gene-Lifestyle Interactions in Dental Endpoints; GWAS, genome-wide association study; HbA1c, hemoglobin A_1C; HCHS/SOL, Hispanic Community Health Study/Study of Latinos; HDL, high-density lipoprotein; HIP, hip circumference; HL, Hispanic/Latino individuals; JHS, Jackson Heart Study; LD, Linkage Disequilibrium; LDL, low-density lipoprotein; LYM, lymphocytes; MESA, Multi-Ethnic Study of Atherosclerosis; MON, monocytes; MPV, mean platelet volume; NEU, neutrophils; Nteeth, Number of teeth; PAD, peripheral artery disease; PLT, platelets; PP, pulse pressure; PSQI, Pittsburgh Sleep Quality Index; RMSSD, root mean squared difference in successive; normal-to-normal inter-beat intervals; SBP, systolic blood pressure; SDNN, standard deviation of normal-to-normal inter-beat intervals; SVE, supraventricular ectopy; TCL, total cholesterol; TIBC, total iron binding capacity; TMD, temporomandibular disorder; TGL, triglycerides; UKB, UK Biobank; VE, ventricular ectopy; WBC, white blood cells; WC, waist circumference; WHI, Women's Health Initiative; WHR, waist-to-hip ratio.

specific region, taking values of 0, 1, or 2, inherited from a given ancestral population) with a trait. Remaining challenges with admixture mapping include the identification of the causal genetic variant(s) that drive the local ancestry associations. Findings from admixture mapping analyses of blood pressure,[32] lipids,[68] pulmonary,[52] obstructive sleep apnea,[33] kidney,[31,69] and metabolites[70] in HCHS/SOL are summarized in Table 2.

Polygenic risk scores

With the rise of GWAS literature and the availability of summary statistics of genetic variant-trait associations, studies employing genetic/polygenic risk scores (GRSs/PRSs), also called PGSs (polygenic scores) have become increasingly common. There is recognition of the potential use of these risk scores for early screening of highly prevalent diseases, including cardiovascular disease, mental health disorders, and cancers.[71–73] Early work in HCHS/SOL, similar to other studies, typically focused on PRSs constructed based on a handful (tens) of genetic variants, such as genome-wide significant variants reported in other studies (typically of European ancestry individuals), tried to optimize variants within genomic regions to be more appropriate for Hispanic/Latinos, or used variants that explicitly generalized in single-variant association testing to Hispanic/Latinos.[74–77] As the field developed, PRSs based on genome-wide clump and thresholding approaches[39,78–80] and later based on Bayesian methods such as LDPred,[81–83] PRS-CS,[84] etc., and PRS summations, and importantly, leveraging summary statistics from multiple GWASs,[85–89] have been applied. Latter types of PRSs typically explained higher proportions of the trait variance, and increase generalizability of PRS to HCHS/SOL. For example, a PRS for estimated glomerular filtration rate constructed from European ancestry and multi-ethnic studies was developed.[85] A combined sum, multi-ethnic PRS, was strongly associated with prevalent CKD and incident CKD in the HCHS/SOL cohort. Table 3 summarizes analytic approaches used and findings from studies using PRSs in HCHS/SOL. One can see the evolution of methods as described above, starting with a limited number of SNPs, clump and threshold, and Bayesian methods, PRS summations, and use of transcriptomic genetic prediction models later on. Traits investigated including anthropometric traits, and chronic diseases, including kidney disease, cardiovascular disease, T2D, and cognitive outcomes.[39,47,74–78,80,82,83,86–93] Overall, HCHS/SOL both alone and in collaboration with large, racially/ethnically and ancestrally diverse consortia has made marked progress toward the understanding of polygenic risk and the generalizability of other ancestry-derived instruments in other ancestrally diverse populations. In early studies, i.e., using PRS based on GWAS of relatively low sample sizes (e.g., up to 200,000 individuals) and based on European ancestries, GWASs performed worse in Hispanic/Latino populations compared with the population of European ancestries, but ongoing initiatives of HCHS/SOL and improvement of availability of large GWASs from studies of diverse populations are poised to continue optimizing PRS utilization across populations.

Fine mapping

SNPs identified in GWAS analyses associated with a trait of interest are often in linkage disequilibrium (LD) with other SNPs in the region, rendering the identification of a causal SNPs for functional studies difficult. Fine-mapping is a method developed to address this issue by identifying credible sets of loci that are in LD for that association, typically using Bayesian statistical techniques. Initially fine-mapping assumed a single causal variant at the locus, usually one with the strongest p value, since then it has been shown that, biologically, multiple causal variants often exist. Methods such as CAVIAR (using individual genotypes data) and FINEMAP (using summary data) were developed to accommodate this hypothesis implementing a stochastic search for causal configurations in a given credible set with a given α threshold. HCHS/SOL investigators, along with PAGE and other consortia collaborations, have made significant contributions to the literature on fine-mapping SNPs associated with traits ranging from anthropometric measures to reproductive outcomes.[94,95] Studies meta-analyzed cohorts in PAGE using the trans-ancestral Metabo-chip data that include genotyped information from HCHS/SOL to fine-map variants for traits such as body mass index (BMI), blood pressure, age at menopause and menarche, and more. A study fine-mapped loci previously associated with BMI and identified novel secondary signals near the genes *FTO, LYPLAL1, COBLL1, IRS1, SLC39A8, TFAP2B,* and *STK33/TRIM66*.[96] Similarly, a study aiming to fine-map blood pressure-associated SNPs in PAGE data identified "putative causal markers" for the genes *FES/FURIN* and *CLCN6/MTHFR*.[97] Other studies also identified putative causal signals for lipid-related traits, BMI, height, waist-to-hip ratio, C-reactive protein (CRP), electrocardiogram PR interval, red blood cell traits, and kidney function.[22,54,98–101] Fernández-Rhodes and colleagues observed that they were able to identify secondary signals for reproductive traits after using modified random effects meta-analysis (as employed in MetaSoft) combined across four race/ethnic groups. This yielded one array-wide significant locus for *CUX2* that was in low LD with known age at menarche or other cardiometabolic traits, and two array-wide significant loci for age at natural menopause (*FRMD5* and *GPRC5B*).[102]

Inclusion of data with strong primary signals and diverse genomes can augment the search for causal configurations because allele frequencies and LD patterns vary across ancestries. Therefore, the inclusion of datasets like HCHS/SOL stands to increase the power and resolution of the findings. Wojcik and colleagues identified 38 secondary signals for previously associated SNPs across 26

Table 2. Summary of admixture mapping studies

Phenotype/outcome	Primary findings	Localized SNPs	Replication	Reference(s)
Blood pressure traits	Amerindian genetic ancestry association regions on 6p21.31, 11q13.4, 17q25.3. African genetic ancestry association region on 6p12.3.	Multiple variants jointly explaining the association region 6p21.31. Other regions without identified SNPs explaining or not fully explaining the association.	rs138977532 in 6p21.31 associated with BP traits in Pima Indians.	Sofer et al.[32]
Lipid measures	No admixture mapping association regions detected, suggesting similar frequencies of top lipid loci in ancestral populations.	–	–	Andaleon et al.[68]
Pulmonary outcomes	Amerindian ancestry with forced expiratory volume in 1 s (FEV_1) in 7p21.2 region.	rs41331850	rs41331850 association with FEV_1 was replicated in the UK Biobank.	Burkart & Sofer et al.[52]
Sleep apnea	Three association regions where African, Amerindian, or European ancestry levels were associated with indices of sleep apnea.	rs6750391 at *2q37* associated with apnea hypopnea index (AHI). rs57403733 at 18q21 was associated with AHI and with percent sleep time with less than 90% oxyhemoglobin saturation. Two SNPs jointly, partially, explained association region on 16q12-21 with respiratory event duration.	Replication attempt at the regional level, but not statistically significant replication signal considering multiple testing.	Wang et al.[33]
Kidney traits	Three association regions with albuminuria driven by Amerindian, African, and European ancestries. Two association regions of European ancestry and one of African ancestry levels with chronic kidney disease (CKD).	Albuminuria association region with Amerindian ancestry on chromosome 2 spanning q11.2–14.1 explained by rs116907128. No SNPs suggested for other albuminuria association regions. No SNPs were detected as candidates to explain the admixture mapping associations with CKD.	rs116907128 association with albuminuria replicated in Pima Indians. Two admixture mapping association regions with CKD were replicated in local ancestry association analysis in Black individuals from the Women's Health Initiative.	Brown et al.,[31] Horimoto et al.[69]
Metabolites panel	78 metabolites had 484 association regions with ancestry counts. Long genomic region at chromosomes 11 with many metabolite associations with Amerindian ancestry, primarily with lipid metabolites, suggesting evolutionary selection related to diet. Long region on chromosome 2 with eight N-acetylated amino acid metabolites associated with African ancestry.	Multiple SNPs, primarily intronic to the *FADS1* and *FADS2* genes that are involved in fatty acid metabolism, explained chromosome 11 associations. Seven SNPs intronic to *ALMS1/ALMS1P* explained the chromosome 2 associations.	More than 50% of admixture mapping associations replicated in a held-out dataset from HCHS/SOL, and all association estimates in the replication sample had the same direction of associations as that of the discovery sample.	Reynolds et al.[70]

AHI, apnea hypopnea index; BP, blood pressure; CKD, chronic kidney disease; FEV_1, forced expiratory volume in 1 s; HCHS/SOL, Hispanic Community Health Study/Study of Latinos; SNP, single nucleotide polymorphism.

Table 3. Summary of polygenic risk score studies

PRS trait	PRS type	Analysis and findings	Reference
**HCHS/SOL only**			
BMI	Sum of 97 BMI-increasing alleles reported in European ancestry population GWAS.	BMI PRS is associated with clinical obesity.	Guo et al.[74]
BMI	Based on 97 BMI SNPs, computed an overall score, and scores based on SNPs related and unrelated to the central nervous system (CNS).	Individuals with higher physical activity levels or less sedentary time had weaker PRS-BMI associations with anthropometric traits for compared with those having unfavorable activity patterns. These patterns were driven by the CNS BMI GRS component.	Moon et al.[75]
Depression	Using, separately, three published GWAS of depression in European ancestries individuals, constructed PRS summing trait-increasing alleles from independent SNPs based on a few *p* value thresholds.	PRS based on PGC MDD2 and 23andMe meta-analysis (*p* value thresholds <0.001) were associated with depression symptoms scores in HCHS/SOL. PRS based on smaller GWAS were not.	Dunn et al.[39]
Height	Primary PRS: unweighted sum of known height-increasing alleles. Secondary PRS: sum of 604 height-increasing alleles from lead SNPs at height association regions.	Height PRSs were evaluated for association and mediation (with standing height as mediator) with cardiometabolic and pulmonary phenotypes. The PRSs associated with lung function, total cholesterol, and oral glucose-tolerance insulin levels. Higher height PRSs had both direct and mediated effects on reduced lung function.	Sofer & Moon et al.[76]
Cardiometabolic and blood count phenotypes	Methodological paper comparing approaches for variant selection and weights when using summary statistics from a large European ancestry GWAS and a small GWAS of the target ancestry. PRSs were based on clumping and thresholding.	Selecting variants based on a large European ancestry GWAS often performed well, but it was better to use weights from either a meta-analysis of the two GWASs or from the GWAS based on similar ancestry to the target population.	Grinde et al.[89]
Height	Sum or weighted sum of 1,078 lead SNP alleles at least 1 Mb away from each other.	PRS was associated with height across birth year and ancestry stratifications.	Spear et al.[90]
Cognitive traits and educational attainment	Clump and threshold based on summary statistics of various GWAS of traits associated with cognitive aging. PRSs were tested for association with available cognitive tests scores.	PRS for cognitive traits and PRS for educational attainment was associated with cognitive traits in HCHS/SOL, but explained very little of trait variances.	Jian et al.[47]
BMI	BMI PRS was developed using LDPred and GIANT + UKB European ancestries GWAS meta-analysis.	BMI PRS had larger effect size estimates for BMI in females compared to males. Early age at immigration to the United States and healthy diet were associated with higher BMI PRS effects on BMI and WHR in a sex-specific manner.	McArdle et al.[83]
BMI	Clump and threshold based on summary statistics of European ancestries individuals: UKB and GIANT meta-analysis of BMI GWASs.	BMI PRS and chronic stress were associated with BMI and obesity, but there was no evidence of interaction.	Isasi et al.[80]

(*Continued on next page*)

Table 3. *Continued*

PRS trait	PRS type	Analysis and findings	Reference
OSA, cardiometabolic and pulmonary phenotypes	PRSs for OSA and for genetically correlated traits were constructed using LDPred2 based on mostly multi-ethnic GWAS. Associations with their intended traits were first verified, PRS associations were estimated with genetically correlated traits to their intended trait.	PRSs were associated with their intended traits. PRSs for anthropometric, blood pressure, glycemic traits, and insomnia, were associated with OSA. OSA PRS was associated with BMI, WHR, and glycemic traits, but only with WHR and HbA1c when adjusting for BMI.	Zhang et al.[82]; PGP000456
Sleep phenotypes	Clump and threshold using PRSice based on European ancestry GWAS. Sleep PRSs were used in association analyses with global cognitive phenotypes related to aging.	Higher genetic liability to insomnia and for daytime sleepiness were associated with MCI and global cognitive function. Genetic liability for sleep duration interacted with self-reported sleep duration in association with MCI and reduction in global cognitive function.	Zhang et al.[82]
Hypertension	Sum of 10 HTN risk alleles that were generalized to Hispanic/Latino individuals in a published GWAS. Evaluated in association with HTN as main effect and interaction with chronic stress.	Both the PRS and chronic stress were associated with hypertension, but there was no evidence of interaction effect.	Preudhomme et al.[77]
Kidney function (eGFR)	Clump and threshold using PRSice and PRS-CS based on European ancestry and multi-ancestry GWAS. PRS combined sum was trained in WHI. PRSs were evaluated for association with eGFR, CKD, and hypertension.	PRSs were associated with eGFR, prevalent and incident CKD. Sum of PRS based on two GWASs performed better than the individual PRSs.	Zhou et al.[85]
BMI	Sum of 103 BMI-increasing Hispanic/Latino "best marker" SNPs (MAF>1%, high imputation quality, low heterogeneity across Hispanic backgrounds) from BMI association regions.	BMI PRS association with BMI varied by levels of acculturation to the United States, and by sex, with stronger associations in females and in highly acculturated individuals.	Fernández-Rhodes et al.[91]; PGP000456
Alzheimer disease	PRSs were developed based on summary statistics from multiple GWASs of AD, using PRSice and PRS-CS. PRSs were evaluated individually and as PRS combinations (sums) in MGB Biobank AD individuals, then used in HCHS/SOL.	A sum of AD PRSs was associated with MCI. PRSs that included the *APOE* gene region had stronger associations than PRSs without, even though *APOE* alleles were not associated with MCI.	Sofer & Kurniansyah et al.[88]; PGP000503
**PAGE/TOPMed**			
Lipids	Weighted sums of independent lipid-associated alleles with combination of weights and of variants selection based on European ancestry (GLGC) GWAS and/or PAGE multi-ethnic analysis.	PRSs that used GLGC top variants and weighted by PAGE-estimated effect sizes tended to explain higher proportions of variance. For triglycerides, using PAGE to also prioritize variants within association regions improved variance explained.	Hu et al.[92]

(*Continued on next page*)

Table 3. *Continued*

PRS trait	PRS type	Analysis and findings	Reference
COPD and lung function	Developed and compared genome-wide significant and clump and threshold method applied on a European ancestry GWAS, and PTRSs summing predicted gene expression for a few tissues. A few approaches were compared for inclusion genes with genetically predicted gene expression in the PTRSs. The best performing risk score in the training dataset was evaluated in an independent dataset.	PRSs based on genome-wide significant variants performed best in non-Hispanic White individuals. However, PTRSs performed better in African American individuals.	Hu et al.[93]; PGP000348
Hypertension	PRSs for SBP, DBP, and HTN were developed using summary statistics from a multi-ethnic GWAS and clump and threshold approach. *P*value threshold was selected by minimizing the coefficient of variation over subsets of the training dataset. Summation weights of SBP, DBP, and HTN PRSs were computed over the training dataset.	The final HTN-PRS was a weighted sum of SBP, DBP, and HTN PRSs. It was associated with 4–6 years of incident hypertension across adulthood. Individuals stratified by PRS quartile had different trajectories of HTN development.	Kurniansyah et al.[86]; PGP000531
Blood pressure phenotypes	PRSs were developed using ancestry-specific and multi-ethnic GWAS summary statistics and using clump and threshold, LDPred2, PRS-CS, and PRS-CSx. PRS weights for PRS-CSx PRSs were computed in the MGB Biobank. PRSs were selected in TOPMed. Evaluated in association with BP phenotypes and CVD outcomes in All of Us.	Best performing PRSs were those developed using PRS-CSx. In All of Us, PRSs explained ~1.2% (Black) to ~4% (Hispanic/Latino) residual trait variance, after accounting for age, sex at birth, BMI, smoking, and genetic ancestry PCs. PRS effect sizes and PVEs differed by strata of obesity, age, and sex at birth.	Kurniansyah & Goodman et al.[87]; PGP000510

AD, Alzheimer disease; *APOE*, apolipoprotein E; BMI, body mass index; BP, blood pressure; CKD, chronic kidney disease; COPD, chronic obstructive pulmonary disease; CVD, cardiovascular disease; DBP, diastolic blood pressure; eGFR, estimated glomerular filtration rate; GLGC, global lipids genetic consortium; HbA1c, hemoglobin A1c; HTN, hypertension; LD, linkage disequilibrium; MAF, minor allele frequency; MCI, mild cognitive impairment; MDD, major depressive disorder; MGB, Mass General Brigham; OSA, obstructive sleep apnea; PAGE, Population Architecture using Genomics; PC, principal component; PGC, Psychiatric Genomics Consortium; PTRS, polygenic transcriptomics risk score; PVE, percent variance explained; SBP, systolic blood pressure; SNP, single nucleotide polymorphism; TOPMed, *trans*-omics in precision medicine; WHI, Women's Health Initiative; WHR, waist-to-hip ratio.

harmonized phenotypes and observed novel loci for all phenotypes.[103] Additionally, they observed that although further addition of European background samples increased the amount of variance explained in the phenotype of European populations, it could exacerbate disparities for non-European populations due to the diminished representation of other ancestries and reduce applicability in other ancestries. Another study demonstrated the advantage of adding diverse samples in genetic studies by examining fine-mapping of SNPs after trans-ancestral meta-analysis; it was observed that the inclusion of samples from various genetic ancestry groups refined 67% of known loci where the finemapped 99% credible set included fewer SNPs.[92] They discovered and validated five novel lipid loci and observed that adding PAGE minority samples to GLGC data reduced the number of SNPs included in the 99% credible set from 2,000 to 1 (rs3780181) in the *VLDLR* region. Using PAGE data for fine-mapping glycemic trait-associated SNPs, a study yielded ~72.5% reduction in putative interval of interest and identification of a region with promoter and enhancer sequences in the functional analyses.[104] A study published by Downie et al. analyzed SNPs associated with glycemic traits and observed a cumulative of five novel loci associated with fasting glucose and/or fasting insulin and also identified *LRRC37A5P*, which is a rare African ancestry-specific fasting glucose locus.[95] Their fine-mapping analyses consistently predicted several credible sets near the novel loci identified, further strengthening the rationale for the inclusion of ethnically and racially diverse samples in genetics studies, especially from Hispanic/Latino and African American populations, which shoulder a disproportionate burden of hyperglycemia and cardiometabolic risk. Diverse sample inclusion improves the identification of putative causal loci, as shown by studies conducted using HCHS/SOL data to fine-mapping previously identified variants and is an integral initiative in furthering precision health.

Mendelian randomization

Genetic variants can be major predictors of the body's response to some modifiable factors and environmental exposures. Mendelian randomization (MR) utilizes genetic variants as instrumental variables for these modifiable factors. This method increases causal interpretation as temporality is established and avoids major confounding factors. MR can be conducted using data from either one sample in which the associations between the variants, exposure, and outcome are measured within the same individuals or from two samples in which commonly the genotype-phenotype associations are measured in one sample, and the exposure associations are estimated in a second sample.[105] Each method has strengths, but the two-sample method's major limitation is when the population structure between the samples is not comparable (i.e., genotype-phenotype associations are derived in a European sample while the exposure-outcome associations are estimated in HCHS/SOL). Single-sample MR does not have this limitation, but evaluation of genotype, exposure, and outcome associations within the same sample requires a large sample size with adequate statistical power. As a result of these limitations, most MR studies using HCHS/SOL were two-sample and required validation of the instrumental variants using algorithms controlling for ancestral background, cryptic relatedness, and sample clustering.[106] As a richly genotyped and phenotyped cohort, HCHS/SOL has been used to conduct many MR studies. For instance, the body's ability to metabolize arsenic has known genetic predictors and a few studies using HCHS/SOL have evaluated the impact of arsenic metabolism on disease outcomes using two-sample MR. First, among study participants with high rice consumption, a proxy for increased dietary arsenic exposure, inefficient arsenic metabolism indicated by genetic variants in arsenic metabolizing genes were found to be associated with higher systolic and diastolic blood pressure.[106] Notably, Hispanic/Latino individuals in the United States have relatively high rice consumption, making this population useful to study this issue. Using the same PRS instrument for arsenic metabolizing genes, inefficient arsenic metabolism was also associated with an increased risk of asthma, lower pulmonary forced vital capacity, and reduced peak expiratory flow rate.[107] Metabolomics, or the study of small molecules or metabolites that either are introduced to the body from exogenous sources (e.g., foods, environmental toxins) or produced endogenously, also has genetic predictors. HCHS/SOL researchers have assessed both associations with metabolites and asthma and cognition. First, Lee et al. found a significant association between the metabolite 1-arachidonoyl-GPA (20:4) and asthma.[108] Using two-sample MR with the variant rs28456, they determined the association to be potentially causal as it was conserved when using the genetic instrument as the exposure. Furthermore, Granot-Hershkovitz et al. extracted SNPs from previously conducted GWASs for metabolites found to be associated with cognitive dysfunction.[109] Using two-sample MR, they found weak evidence of causal associations between the metabolites and cognition. Last, evidence for causal associations between two disease states can be determined using MR. Using variants associated with cardiovascular and pulmonary disease phenotypes, HCHS/SOL researchers used two-sample MR to assess the directionality of the associations between cardiovascular and pulmonary disease with obstructive sleep apnea.[82] They found that cardiovascular disease (CVD) risk factors, including BMI, waist-to-hip ratio, blood pressure, and hemoglobin A1c, are likely the factors that explain the association between CVD and obstructive sleep apnea. Overall, these studies conducted using the HCHS/SOL have provided vital information for improving causal inference for several modifiable disease risk factors.

Gene by lifestyle interactions

While an individual's underlying genetics are generally non-modifiable, there is evidence that genetic associations can be influenced by lifestyle factors or environmental exposures. Gene-by-environment (GxE) interaction studies assess the associations between genetic variants and outcomes in the presence or absence of other exposures to identify modifiable factors to attenuate genetic risks. In this regard, it is significant that HCHS/SOL is enriched with extensive lifestyle data, which include both self-reported information as well as data collected using wearables or biomarkers related to diet, exercise habits, and sleep. GxE studies have been conducted at both the single-variant and polygenic levels. At the single-variant level, HCHS/SOL has contributed to multiple analyses from the Cohorts for Heart and Aging Research in Genetic Epidemiology (CHARGE) GxE initiative (for example, Bentley et al.,[110] de Las Fuentes et al.,[111] and de Vries et al.[112]) that successfully identified genetic variants interacting with lifestyle measures in their associations with blood pressure and lipid traits. While high sample sizes are needed for discovery of single-variant interaction associations, PRSs offer a powerful alternative for studying interactions, as they explain larger variance proportions by pooling together information across many single variants, and multiple PRS interaction studies have been done in HCHS/SOL. Obesity is a highly prevalent condition both in the United States and worldwide and commonly occurs within families.[113] As a result, there has been a major push to understand the underlying genetics of obesity and how one's susceptibility may be attenuated or exacerbated by certain exposures. HCHS/SOL studies have also assessed the interaction between polygenic risk for BMI and obesity with physical activity, chronic stress, and Hispanic/Latino acculturation measures. Moon et al. found that those with higher physical activity levels or less sedentary time had weaker associations for established genetic risk factors for obesity, as compared with those having unfavorable activity patterns.[75] McArdle et al. found that those who immigrated to the United States later in life and those with worse diet quality had higher associations between polygenic risk for obesity and increased BMI.[83] Another study studied the potential impact of acculturation to the United States on genetic risk for BMI. The authors found that the estimated effect of BMI PRS on observed BMI was higher among individuals with higher levels of acculturation to the United States, as measured by a few measures, including Spanish language use and time living in the United States.[79] In contrast, Isasi et al. did not detect any statistically significant interaction between the BMI PRS and chronic stress.[80]

GxE studies can also be utilized to assess pharmacogenetics and genetic risk factors for drug side effects. A meta-analysis conducted by Noordam et al. in 2017 assessed genome-wide interaction for tricyclic and tetracyclic antidepressant (TCA) usage with RR and QT interval lengthening on an electrocardiogram; both of which are risk factors for fatal cardiac arrhythmias.[114] They identified several candidate genes that modified the association between TCA usage with prolonged RR and QT interval. An HCHS/SOL-specific study assessed the prevalence of variants that are known to influence the efficacy of response to clopidogrel treatment, which is used to treat acute coronary syndrome. The study found substantial differences in allele frequencies between US Hispanic/Latino and European ancestry populations, suggesting the need to study and adapt treatment recommendations to Hispanic/Latino individuals.[115]

Integration of genetics with metabolomics

Integration of genetics with metabolomics, a study of metabolites measured at scale, has been successfully applied before to identify unique SNPs that regulate metabolites. However, in Hispanic/Latino individuals, a population that is disproportionately burdened with cardiometabolic dysfunction, metabolomics has been applied at large scale only in recent years. In the first assay of metabolomics data, HCHS/SOL performed metabolic profiling of stored serum samples at Metabolon (Durham, NC) with HD4 platform in 2017. A second set of metabolomics profiling was performed in 2021, using the same platform. Studies leveraged the genotyped data and the serum metabolomics data to conduct metabolome-wide genetic studies, as well as admixture mapping, and identified loci associated with blood lipids,[56] long-chain poly unsaturated fatty acids (LC-PUFAs[70]), and amino acids, among others, as well as with microbiota. Studies further linked genetic loci and metabolites to phenotypes such as diseases related to metabolism, age-related macular degeneration,[116] lactose intolerance,[117,118] T2D, and mild cognitive impairment.[119] Data from HCHS/SOL and other Amerindian ancestry populations provide evidence for strong genetic selection and abundant phenotype associations for the *FADS* gene family,[120] which may have played an important role in environmental adaptation during the original founding of the Native American population. A few studies performed multi-step analyses linking metabolites, phenotypes, genetics, and microbiome or RNA sequencing.[117,118,121] Genetics have been used to stratify individuals (e.g., to lactase non-persistent persistent individuals[117]) or as instrumental variables to infer likely direction of causal associations.[121] Table 4 summarizes studies integrating genetics and metabolomics, including the modeling approach used and major findings.

Ethical, legal, and social implications in genetic studies

Beyond studies of genotype-trait associations, HCHS/SOL researchers have discussed various aspects of the ethical and legal implications of genetic research. For the National Institutes of Health genomic data sharing policy,

HCHS/SOL is defined as a "sensitive" study, meaning that summary statistics from GWASs cannot be automatically made publicly available. HCHS/SOL genetic data are available via a data access request from the Database of Genotypes and Phenotypes (dbGaP), and some GWAS summary statistics have been deposited on the HCHS/SOL dbGaP accession. Unlike directly measured, individual-level phenotypes and genotypes, GWAS summary statistics do not provide information on specific participants. However, as described by the members of the TOPMed Ethical, Legal, and Social Issues (ELSI) committee, risks of sharing genome-wide summary statistics are re-identification (inference that a specific person participated in a specific analysis), stigmatization (e.g., due to the association of a high genetic risk to a potentially stigmatizing phenotype to a specific population segment), and consistency with informed consent (downstream use of summary statistics may not be consistent with participant consent).[122]

Among participants of the HCHS/SOL, at baseline, almost all participants consented to the use of genetic data by study investigators, although only 75% granted consent for commercial entities to utilize their genetic information,[123] suggesting that HCHS/SOL participants trust the study itself, while some negatively view the idea that others will profit from their specimen or data. Participant attitudes toward sharing genetic data may be influenced by knowledge and awareness of the uses of genetic testing and genetic research. HCHS/SOL researchers theorized that participation and openness differed by acculturation and immigration statuses within communities.[123] A survey conducted among HCHS/SOL participants found that only 55% were familiar with genetic testing, and on average, US-born participants rated genetic information less valuable than those born outside of the United States.[124]

An essential scientific discussion of high relevance to HCHS/SOL investigators and participants is the use of population descriptors (Box 1). In the past, scientists have often used race as a proxy for human genetic variation—an idea rooted in racism, eugenics, and other harmful forms of scientific discrimination.[125] The usage of ethnoracial terms and population ancestry-based grouping emerged out of convenience and practicality in genomics and has little to do with the elucidation of true biological ancestry or racial/ethnic background.[125] Efforts to correct the interpretation of descent-associated population descriptors have been undertaken in the past 20 years. Yet, there is a lack of clear consensus on what the descriptors should be.[125–128] Guidelines are emerging with an emphasis on careful consideration of the scientific process leading to specific analysis, from participant recruitment and their self-identification, harmonization of race, ethnicity, and genetic ancestry categories, to the elucidation of analysis hypotheses informing the choices of population descriptors and related analysis variables.[125,128] HCHS/SOL researchers have emphasized respect for cultural preferences in the labeling process for the collection of sensitive data such as Hispanic/Latino heritage, nativity, and language preferences. Indeed, for epidemiologic studies, HCHS/SOL recommends that researchers use "heritage" or "background" to describe the diversity of Hispanic/Latino populations in HCHS/SOL. A race variable is not used in most HCHS/SOL analyses, as about half of the participants do not self-identify with a racial category. In genetic analysis, HCHS/SOL investigators debated the use of Hispanic/Latino background. They showed that self-reported Hispanic/Latino background is strongly correlated with genetic patterns (Figure 2), likely due to migration patterns and resulting admixture.[4,129] Consequently, researchers developed "genetic analysis groups" that roughly overlap with groups defined by self-reported Hispanic/Latino background but are genetically more homogeneous. The term "genetic analysis groups" reflects that idea that these groupings are only used for genetic analysis to reduce heterogeneity and therefore increase power for discovering genetic associations but are not scientifically, biologically, or socially meaningful otherwise.[4]

HCHS/SOL researchers have also participated in discussions about the implementation of genetic research findings in clinical practice. The effective translation of genetic findings and implementation of PRS predictions in clinical practice are laden with concerns about PRS validity, their transportability across subpopulations, cost, and provider and patient education.[130] Implementation of genetic risk prediction should also take into consideration outcome prevention and early detection. The use of individual genetic data should also consider appropriate risk stratification and what steps may be undertaken in clinical settings for those who are classified as low risk to effectively monitor the progression in all sub-groups.[131] At the single risk variant level, HCHS/SOL and other large studies have discovered genetic variants that could be very useful in clinical practice including variants associated with benign neutropenia and T2D that are rather common in populations of African descent, rare in populations of European descent, but are found in admixed Hispanic/Latino populations.[25,132] Such variants raise questions about potential screening and risk stratification efforts that may rely on admixture patterns of specific Hispanic/Latino background groups, i.e., by prioritizing screening efforts in individuals self-identifying with a Hispanic/Latino background group that tends to have higher African admixture. This is addressed by ongoing work from HCHS/SOL investigators, and we recognize that such work should be accompanied by ELSI-related discussions, and to the extent possible community participation.[133,134]

Ethics statement

The HCHS/SOL was approved by the institutional review boards (IRBs) at each field center, where all participants gave written informed consent, and by the Non-Biomedical IRB at the University of North Carolina at Chapel Hill, to the HCHS/SOL Data Coordinating Center. All IRBs approving the HCHS/SOL study are Non-Biomedical IRB at the University of North Carolina at

Table 4. Genetics-metabolomics integration studies in HCHS/SOL

Study	Modeling approach	Major findings	Reference
GWAS of 640 metabolites	GWAS of each metabolite, with replication testing of new association regions in independent studies. Follow-up analyses: colocalization analysis with eQTLs from GTEx; MR analysis using newly discovered variants as instrumental variables for metabolites, in association with CHD, FH, and T2D.	Forty-six new loci-metabolite associations, which replicated to ARIC and TwinsUK studies. These findings included loci of endocannabinoids related to drug addiction, steroid-derived molecules, phosphatidylcholines, and others. Follow-up MR analysis identified metabolites causally associated with CHD and T2D.	Feofanova et al.[56]
Admixture mapping of 640 metabolites	Admixture mapping testing the associations of African, European, and Native American local ancestries with metabolite levels, followed by replication in a separate dataset from HCHS/SOL. Follow-up analyses: conditional analyses using genome-wide significant variants from association regions.	232 novel local ancestry region-metabolite associations. Observed metabolites were associated with diet restriction and exposure to pathogens affecting inflammation and other biological processes. Native American local ancestry levels were inversely related to levels of multiple LC-PUFAs.	Reynolds et al.[70]
Integrative analysis of T2D-associated tryptophan metabolites	Association analysis of 11 tryptophan metabolites with T2D in race/ethnic diverse participants from five cohorts. Associated metabolites were examined for associations with host generic variants, dietary factors, gut bacteria, and interactions among them. Mediation analyses linking genetic and dietary factors, gut genera, metabolites, and T2D.	Five tryptophan metabolites associated with incident T2D, including indolepriopionate, which was inversely associated with T2D incidence. The Lactase persistence *LCT* gene variant rs4988235 was associated with serum indolepriopionate and the association was mediated by gut microbiota including *Bifidobacterium*. Higher milk intake was associated with higher levels of *Bifidobacterium* and of indolepriopionate only in lactase non-persistent adults.	Qi et al.[118]
Milk intake, incident T2D, and mediating pathways, among lactase-persistent (LP) and lactase non-persistent (LNP) individuals	Within strata of LNP and LP: Association between dairy intake and incident diabetes; between dairy intake and bacterial species; between dairy intake and metabolites. Follow-up mediation analyses linking milk intake, bacterial species, metabolites, and incident T2D. Replication analyses in UKB and in ARIC.	Milk intake is associated with reduced T2D in LNP but not in LP individuals. The association of milk intake with gut bacteria and with serum metabolites differs between NLP and LP individuals, and gut bacteria often mediates the associations between milk intake with metabolite levels.	Luo et al.[117]
Genetic effects on MCI mediated via the BAIBA metabolite	GWAS of a metabolite risk score (MRS) that is associated with MCI, identification of relevant gene, repeated conditional analyses, and replication of independent SNP associations. Mediation analysis with SNPs, BAIBA, and MCI.	Genetic association region of the MRS is the *AGXT2* gene, having a role in BAIBA metabolism. Some of the SNPs associated with the MRS and with BAIBA show evidence of association with MCI in ARIC. Evidence of BAIBA levels mediating *AGXT2* region genetic effects on MCI.	Granot-Hershkovitz et al.[109]

(*Continued on next page*)

Table 4. ***Continued***

Study	Modeling approach	Major findings	Reference
SNP-metabolite associations in AMD	Compiled a comprehensive database of metabolomic quantitative trait loci (mQTLs) from up to 28,000 participants across different ancestries from 6 cohorts including HCHS/SOL. Used mQTLs to perform MR analysis of metabolites (exposures, each individually) associated with advanced AMD. Performed genetically predicted metabolome-wide association analysis with AMD using metabolite genetic scores in leu of metabolites. Performed Bayesian colocalization and pathway enrichment analyses.	Identified 108 plasma metabolites with possible causal association with AMD. Genetic scores of 155 metabolites were associated with AMD. Suggested 114 causal variants for metabolite and AMD risk. Top gene regions included *APOE*, *ABCA1*, *LIPC*, and *CETP*. Metabolites associated with AMD and their genetic determinants were common to both European and Hispanic populations.	Han et al.[116]
Causal pathways linking SDB, transcript expression, and metabolite levels	Collaborative study of three studies: HCHS/SOL, MESA, and WHI, constructing and validating polygenic scores as "genetic instruments" for transcript expression levels from multiple blood tissues. Next performing association analyses of SDB with transcriptomics, transcriptomics, and finally metabolomics and SDB, suggesting "causal chains" supporting causal directions with association analyses using the genetic instruments.	Association of SDB with gene expression levels differ by blood cell type. The expressin of the Purinergic Receptor P2X 4 (*P2XR4P*) gene expression in peripheral blood mononuclear cells is inversely associated with average oxyhemoglobin during sleep (AvgO2), in a possibly bi-directional relationship. Higher expression of *P2XR4* and lower AvgO2 are associated with higher levels of butyrylcarnitine (C4).	Kurniansyah et al.[121]

ARIC, atherosclerosis risk in communities; BAIBA, beta aminoisobutyric acid; CHD, coronary artery disease; eQTL, expression quantitative trait locus; GTEx, genotype-tissue expression project; GWAS, genome-wide association study; LC-PUFA, long-chain polyunsaturated fatty acid; MAD, age-related macular degeneration; MCI, Mild cognitive impairment; MR, Mendelian randomization; MRS, metabolite risk score; mQTL, metabolite quantitative trait locus; SDB, sleep disordered breathing; SNP, single nucleotide polymorphism; T2D, type 2 diabetes; UKB, United Kingdom biobank; WHI, Women's Health Initiative.

Box 2. Future challenges of genetic analyses in HCHS/SOL

We here highlight a few areas of research that we expect to be tackled by HCHS/SOL investigators in the next few years due to advent of data and technology, as well as the development of research directions based on new understandings of the genetic architecture of complex traits.

Omics research and its integration

- Additional omics data typing in progress:
  - Serum metabolomics – most consenting visit 1 and 2 participants.
  - Whole-blood methylation – most consenting visit 1 and 2 participants.
  - Whole-blood RNA sequencing – estimated 7,700 visit 1 participants.
  - Plasma proteomics – estimated 2,000 visit 1 participants.
- Datasets will undergo rigorous quality control.
- Offers comprehensive view of biological processes for health and disease beyond existing genetic measures.
- HCHS/SOL contributes to studies of the impact of genetic variation relative to highly represented populations and to studies of associations of diverse environmental and lifestyle exposures.
- Omics will allow for improvement in identification and fine mapping of causal variants[136,137] and PRS in admixed populations.[138]

Machine and deep learning

- Methodology used for all types of genetic analyses, including
  - Predictive modeling of disease development[139,140]
  - Classification of variants into categories of potential impact[141]
  - Implementation of pattern recognition, e.g., identification of disease subtypes,[142] for diagnosis[143] and model interpretation.[144]
- Integration of omics data and multiple data types including imaging, electrophysiological signals, and environmental exposures.
- Data may need to be harmonized and aggregated across multiple studies as HCHS/SOL is limited in data size for machine learning (ML)/deep learning (DL) models, and models may need to be calibrated.[145]

Population genetics analyses in relation to health and disease phenotypes

- Deviations from the uniform distribution of local ancestry proportions for a specific genomic region indicates an evolutionary advantage for a genetic variant often due to immunity or resistance to disease.[146,147]
- Current literature suggests that exposure to pathogens drives genetic selective pressures.[148]
- Data from HCHS/SOL will help evaluate the roles of diet, assortative mating, and potentially other environmental exposures on genetic selection[149] and distinguish them from genetic drift.[150]

Gene-environment interactions

- Some genetic effects manifest under specific exposures.[135]
- HCHS/SOL studies GxE interactions for both lifestyle and environmental exposures using the comprehensive and harmonized data collection in this multicenter study.
- Identification of GxE interactions can be modeled and detected at the variant,[117] at the PRS,[91] or at the pathway level.[151]
- ML/DL models may be especially useful in unveiling GxE interactions.[152]

Precision medicine in admixed individuals

- Aims to develop interventions to individual genetic, environmental, and lifestyle characteristics.
- Omics, population genetics, ML/DL, and gene-environment interactions can be leveraged into the implementation of precision medicine in Hispanic/Latino individuals.
- Special considerations for admixed individuals can assess whether utilization of ancestral information at the global and local levels is useful independent of directly measured genetic variants.
- Can determine if genetic variants associated with specific health measures, such as the Duffy null variant associated with benign neutropenia and common in African genetic ancestries,[153] are useful for making specific medical decisions.

Chapel Hill, Chapel Hill, NC; Einstein IRB at the Albert Einstein College of Medicine of Yeshiva University, Bronx, NY; IRB at Office for the Protection of Research Subjects (OPRS), University of Illinois at Chicago, Chicago, IL; Human Subject Research Office, University of Miami, Miami, FL; and IRB of San Diego State University, San Diego, CA. All methods and analyses of HCHS/SOL participants' materials and data were carried out in accordance with human subject research guidelines and regulations.

Summary and conclusion

The HCHS/SOL has made major contributions to genetic research and genetic medicine among Hispanic/Latino individuals in the United States. Since the inception of the HCHS/SOL cohort, genetic researchers have published over 75 research articles from HCHS/SOL addressing genetic risk, methodology, bioethics, diversity, and inclusion. Statistical methodology motivated by HCHS/SOL addressed several limitations in genetic research, especially among non-European populations while reducing type 1 error, increasing statistical power, and more. Furthermore, while HCHS/SOL has made important independent contributions to genetic research, its participation in other genetic consortia has enabled further research progress.

HCHS/SOL was uniquely designed to enable the study of health within, as well as the heterogeneity between, Hispanic/Latino background groups. In terms of genetic associations, while we do see heterogeneity between Hispanic/Latino background groups arising from differences in admixture patterns and allele frequency differences between the ancestral populations, there is little evidence for heterogeneity in the genotype-phenotype associations themselves. Currently, HCHS/SOL is in the process of engaging in other omics-based research, including metabolomics, microbiome, RNA sequencing, and epigenetics. We expect that these data will lead to important insights about the role of genetics, admixture, and the environment, in Hispanic/Latino individuals and human health in general. We further expect that omics data will facilitate studies of how environmental effects, including those of socioeconomic status, health care access, education, and economic opportunities, potentially mediate and moderate genetic effects.[135] Box 2 summarizes these and additional challenges that HCHS/SOL investigators will face in the next several years. HCHS/SOL continues to contribute to conversations about the ethical, legal, and clinical impacts of genetic research. Future studies are needed to incorporate the implementation of genetic medicine in admixed populations, at both the individual and the provider levels, to further precision medicine.

Data and code availability

HCHS/SOL data are available through application to the database of genotypes and phenotypes (dbGaP) accession phs000810, or via a data use agreement with the HCHS/SOL Data Coordinating Center (DCC) at the University of North Carolina at Chapel Hill, see collaborators' website: https://sites.cscc.unc.edu/hchs/. No code was specifically developed for this work.

Acknowledgments

The authors thank the staff and participants of the Hispanic Community Health Study/Study of Latinos (HCHS/SOL) for their important contributions to the HCHS/SOL studies. The HCHS/SOL is a collaborative study supported by contracts from the National Heart, Lung, and Blood Institute (NHLBI) to the University of North Carolina (HHSN268201300001I/N01-HC-65233), University of Miami (HHSN268201300004I/N01-HC-65234), Albert Einstein College of Medicine (HHSN268201300002I/N01-HC-65235), University of Illinois at Chicago (HHSN268201300003I/N01- HC-65236 Northwestern Univ), and San Diego State University (HHSN268201300005I/N01-HC-65237). The following Institutes/Centers/Offices have contributed to the HCHS/SOL through a transfer of funds to the NHLBI: National Institute on Minority Health and Health Disparities, National Institute on Deafness and Other Communication Disorders, National Institute of Dental and Craniofacial Research, National Institute of Diabetes and Digestive and Kidney Diseases, National Institute of Neurological Disorders and Stroke, NIH Institution-Office of Dietary Supplements. R01HL163262 supported L.F.-R.; F30ES033510 supported M.C.W.; NIH/NHLBI HHSN 72N92019D00010 supported K.M.P. K.E.N. was supported by grants R01HL142302, R01HL151152, R01DK122503, R01HD057194, R01HG010297, R01HL143885, R01HL163262. T.S. was supported by R01HL161012, R01AG07

5758, R01AG080598. American Heart Association grant # 24PRE1193934/HRIDYA RAO/2024 and National Center for Advancing Translational Sciences, grant TL1 TR002016 and grant UL1 TR002014 supported H.C.R. The content is solely the responsibility of the authors and does not necessarily represent the official views of the NIH. The authors are thankful for the excellent review comments from Dr. Eduardo Tarazona-Santos and the anonymous reviewer.

Declaration of interests

The authors declare no competing interests.

Supplemental information

Supplemental information can be found online at https://doi.org/10.1016/j.xhgg.2024.100376.

References

1. Popejoy, A.B., and Fullerton, S.M. (2016). Genomics is failing on diversity. Nature *538*, 161–164.
2. Passel, J.S., Lopez, M.H., and D'Vera, C. (2011). Census 2010: 50 Million Latinos Hispanics Account for More than Half of Nation's Growth in Past Decade, https://www.pewresearch.org/short-reads/2022/02/03/u-s-hispanic-population-continued-its-geographic-spread-in-the-2010s/.
3. Lavange, L.M., Kalsbeek, W.D., Sorlie, P.D., Avilés-Santa, L.M., Kaplan, R.C., Barnhart, J., Liu, K., Giachello, A., Lee, D.J., Ryan, J., et al. (2010). Sample design and cohort selection in the Hispanic Community Health Study/Study of Latinos. Ann. Epidemiol. *20*, 642–649.
4. Conomos, M.P., Laurie, C.A., Stilp, A.M., Gogarten, S.M., McHugh, C.P., Nelson, S.C., Sofer, T., Fernández-Rhodes, L., Justice, A.E., Graff, M., et al. (2016). Genetic Diversity and Association Studies in US Hispanic/Latino Populations: Applications in the Hispanic Community Health Study/Study of Latinos. Am. J. Hum. Genet. *98*, 165–184.
5. Mills, M.C., and Rahal, C. (2020). The GWAS Diversity Monitor tracks diversity by disease in real time. Nat. Genet. *52*, 242–243.
6. Sirugo, G., Williams, S.M., and Tishkoff, S.A. (2019). The Missing Diversity in Human Genetic Studies. Cell *177*, 1080.
7. Buniello, A., MacArthur, J.A.L., Cerezo, M., Harris, L.W., Hayhurst, J., Malangone, C., McMahon, A., Morales, J., Mountjoy, E., Sollis, E., et al. (2019). The NHGRI-EBI GWAS Catalog of published genome-wide association studies, targeted arrays and summary statistics 2019. Nucleic Acids Res. *47*, D1005–D1012.
8. Pirzada, A., Cai, J., Heiss, G., Sotres-Alvarez, D., Gallo, L.C., Youngblood, M.E., Avilés-Santa, M.L., González, H.M., Isasi, C.R., Kaplan, R., et al. (2023). Evolving Science on Cardiovascular Disease Among Hispanic/Latino Adults: JACC International. J. Am. Coll. Cardiol. *81*, 1505–1520.
9. Clarke, L., Zheng-Bradley, X., Smith, R., Kulesha, E., Xiao, C., Toneva, I., Vaughan, B., Preuss, D., Leinonen, R., Shumway, M., et al. (2012). The 1000 Genomes Project: data management and community access. Nat. Methods *9*, 459–462.
10. Bien, S.A., Wojcik, G.L., Hodonsky, C.J., Gignoux, C.R., Cheng, I., Matise, T.C., Peters, U., Kenny, E.E., and North, K.E. (2019). The Future of Genomic Studies Must Be Globally Representative: Perspectives from PAGE. Annu. Rev. Genomics Hum. Genet. *20*, 181–200.
11. Kowalski, M.H., Qian, H., Hou, Z., Rosen, J.D., Tapia, A.L., Shan, Y., Jain, D., Argos, M., Arnett, D.K., Avery, C., et al. (2019). Use of >100,000 NHLBI Trans-Omics for Precision Medicine (TOPMed) Consortium whole genome sequences improves imputation quality and detection of rare variant associations in admixed African and Hispanic/Latino populations. PLoS Genet. *15*, e1008500.
12. Patterson, N., Price, A.L., and Reich, D. (2006). Population structure and eigenanalysis. PLoS Genet. *2*, e190.
13. Cardon, L.R., and Palmer, L.J. (2003). Population stratification and spurious allelic association. Lancet *361*, 598–604.
14. Kaplan, J.M., and Fullerton, S.M. (2022). Polygenic risk, population structure and ongoing difficulties with race in human genetics. Philos. Trans. R. Soc. Lond. B Biol. Sci. *377*, 20200427.
15. Freedman, M.L., Reich, D., Penney, K.L., McDonald, G.J., Mignault, A.A., Patterson, N., Gabriel, S.B., Topol, E.J., Smoller, J.W., Pato, C.N., et al. (2004). Assessing the impact of population stratification on genetic association studies. Nat. Genet. *36*, 388–393.
16. Hellwege, J.N., Keaton, J.M., Giri, A., Gao, X., Velez Edwards, D.R., and Edwards, T.L. (2017). Population Stratification in Genetic Association Studies. Curr. Protoc. Hum. Genet. *95*, 1.22.1–1.22.23.
17. Musharoff, S., Park, D., Dahl, A., Galanter, J., Liu, X., Huntsman, S., Eng, C., Burchard, E.G., Ayroles, J.F., and Zaitlen, N. (2018). Existence and implications of population variance structure. Preprint at bioRxiv. https://doi.org/10.1101/439661.
18. Sofer, T., Zheng, X., Laurie, C.A., Gogarten, S.M., Brody, J.A., Conomos, M.P., Bis, J.C., Thornton, T.A., Szpiro, A., O'Connell, J.R., et al. (2021). Variant-specific inflation factors for assessing population stratification at the phenotypic variance level. Nat. Commun. *12*, 3506.
19. Sofer, T., Shaffer, J.R., Graff, M., Qi, Q., Stilp, A.M., Gogarten, S.M., North, K.E., Isasi, C.R., Laurie, C.C., and Szpiro, A.A. (2016). Meta-Analysis of Genome-Wide Association Studies with Correlated Individuals: Application to the Hispanic Community Health Study/Study of Latinos (HCHS/SOL). Genet. Epidemiol. *40*, 492–501.
20. Gogarten, S.M., Sofer, T., Chen, H., Yu, C., Brody, J.A., Thornton, T.A., Rice, K.M., and Conomos, M.P. (2019). Genetic association testing using the GENESIS R/Bioconductor package. Bioinformatics *35*, 5346–5348.
21. Chen, H., Wang, C., Conomos, M.P., Stilp, A.M., Li, Z., Sofer, T., Szpiro, A.A., Chen, W., Brehm, J.M., Celedón, J.C., et al. (2016). Control for Population Structure and Relatedness for Binary Traits in Genetic Association Studies via Logistic Mixed Models. Am. J. Hum. Genet. *98*, 653–666.
22. Mägi, R., Horikoshi, M., Sofer, T., Mahajan, A., Kitajima, H., Franceschini, N., and McCarthy, M.I. (2017). Trans-ethnic meta-regression of genome-wide association studies accounting for ancestry increases power for discovery and improves fine-mapping resolution. Hum. Mol. Genet. *26*, 3639–3650.
23. Sofer, T., Heller, R., Bogomolov, M., Avery, C.L., Graff, M., North, K.E., Reiner, A.P., Thornton, T.A., Rice, K., Benjamini, Y., et al. (2017). A powerful statistical framework for generalization testing in GWAS, with application to the HCHS/SOL. Genet. Epidemiol. *41*, 251–258.
24. Justice, A.E., Young, K., Gogarten, S.M., Sofer, T., Graff, M., Love, S.A.M., Wang, Y., Klimentidis, Y.C., Cruz, M., Guo, X., et al. (2021). Genome-wide association study of body

fat distribution traits in Hispanics/Latinos from the HCHS/SOL. Hum. Mol. Genet. *30*, 2190–2204.

25. Qi, Q., Stilp, A.M., Sofer, T., Moon, J.Y., Hidalgo, B., Szpiro, A.A., Wang, T., Ng, M.C.Y., Guo, X., et al.; MEta-analysis of type 2 DIabetes in African Americans MEDIA Consortium (2017). Genetics of Type 2 Diabetes in U.S. Hispanic/Latino Individuals: Results From the Hispanic Community Health Study/Study of Latinos (HCHS/SOL). Diabetes *66*, 1419–1425.
26. Sorlie, P.D., Avilés-Santa, L.M., Wassertheil-Smoller, S., Kaplan, R.C., Daviglus, M.L., Giachello, A.L., Schneiderman, N., Raij, L., Talavera, G., Allison, M., et al. (2010). Design and implementation of the Hispanic Community Health Study/Study of Latinos. Ann. Epidemiol. *20*, 629–641.
27. Sofer, T. (2017). Confidence intervals for heritability via Haseman-Elston regression. Stat. Appl. Genet. Mol. Biol. *16*, 259–273.
28. Elgart, M., Goodman, M.O., Isasi, C., Chen, H., Morrison, A.C., de Vries, P.S., Xu, H., Manichaikul, A.W., Guo, X., Franceschini, N., et al. (2022). Correlations between complex human phenotypes vary by genetic background, gender, and environment. Cell Rep. Med. *3*, 100844.
29. Isasi, C.R., Carnethon, M.R., Ayala, G.X., Arredondo, E., Bangdiwala, S.I., Daviglus, M.L., Delamater, A.M., Eckfeldt, J.H., Perreira, K., Himes, J.H., et al. (2014). The Hispanic Community Children's Health Study/Study of Latino Youth (SOL Youth): design, objectives, and procedures. Ann. Epidemiol. *24*, 29–35.
30. Browning, S.R., Browning, B.L., Daviglus, M.L., Durazo-Arvizu, R.A., Schneiderman, N., Kaplan, R.C., and Laurie, C.C. (2018). Ancestry-specific recent effective population size in the Americas. PLoS Genet. *14*, e1007385.
31. Brown, L.A., Sofer, T., Stilp, A.M., Baier, L.J., Kramer, H.J., Masindova, I., Levy, D., Hanson, R.L., Moncrieft, A.E., Redline, S., et al. (2017). Admixture Mapping Identifies an Amerindian Ancestry Locus Associated with Albuminuria in Hispanics in the United States. J. Am. Soc. Nephrol. *28*, 2211–2220.
32. Sofer, T., Baier, L.J., Browning, S.R., Thornton, T.A., Talavera, G.A., Wassertheil-Smoller, S., Daviglus, M.L., Hanson, R., Kobes, S., Cooper, R.S., et al. (2017). Admixture mapping in the Hispanic Community Health Study/Study of Latinos reveals regions of genetic associations with blood pressure traits. PLoS One *12*, e0188400.
33. Wang, H., Cade, B.E., Sofer, T., Sands, S.A., Chen, H., Browning, S.R., Stilp, A.M., Louie, T.L., Thornton, T.A., Johnson, W.C., et al. (2019). Admixture mapping identifies novel loci for obstructive sleep apnea in Hispanic/Latino Americans. Hum. Mol. Genet. *28*, 675–687.
34. Grinde, K.E., Brown, L.A., Reiner, A.P., Thornton, T.A., and Browning, S.R. (2019). Genome-wide Significance Thresholds for Admixture Mapping Studies. Am. J. Hum. Genet. *104*, 454–465.
35. Zhang, Q.S., Browning, B.L., and Browning, S.R. (2016). ASAFE: ancestry-specific allele frequency estimation. Bioinformatics *32*, 2227–2229.
36. Granot-Hershkovitz, E., Sun, Q., Argos, M., Zhou, H., Lin, X., Browning, S.R., and Sofer, T. (2022). AFA: Ancestry-specific allele frequency estimation in admixed populations: The Hispanic Community Health Study/Study of Latinos. HGG Adv. *3*, 100096.
37. Swenson, B.R., Louie, T., Lin, H.J., Méndez-Giráldez, R., Below, J.E., Laurie, C.C., Kerr, K.F., Highland, H., Thornton, T.A., Ryckman, K.K., et al. (2019). GWAS of QRS duration identifies new loci specific to Hispanic/Latino populations. PLoS One *14*, e0217796.
38. Sanders, A.E., Sofer, T., Wong, Q., Kerr, K.F., Agler, C., Shaffer, J.R., Beck, J.D., Offenbacher, S., Salazar, C.R., North, K.E., et al. (2017). Chronic Periodontitis Genome-wide Association Study in the Hispanic Community Health Study/Study of Latinos. J. Dent. Res. *96*, 64–72.
39. Dunn, E.C., Sofer, T., Wang, M.J., Soare, T.W., Gallo, L.C., Gogarten, S.M., Kerr, K.F., Chen, C.Y., Stein, M.B., Ursano, R.J., et al. (2018). Genome-wide association study of depressive symptoms in the Hispanic Community Health Study/Study of Latinos. J. Psychiatr. Res. *99*, 167–176.
40. Cade, B.E., Chen, H., Stilp, A.M., Gleason, K.J., Sofer, T., Ancoli-Israel, S., Arens, R., Bell, G.I., Below, J.E., Bjonnes, A.C., et al. (2016). Genetic Associations with Obstructive Sleep Apnea Traits in Hispanic/Latino Americans. Am. J. Respir. Crit. Care Med. *194*, 886–897. https://doi.org/10.1164/rccm.201512-2431OC.
41. Morrison, J., Laurie, C.C., Marazita, M.L., Sanders, A.E., Offenbacher, S., Salazar, C.R., Conomos, M.P., Thornton, T., Jain, D., Laurie, C. A., et al. (2016). Genome-wide association study of dental caries in the Hispanic Communities Health Study/Study of Latinos (HCHS/SOL). Hum. Mol. Genet. *25*, 807–816. https://doi.org/10.1093/hmg/ddv506.
42. Schick, U.M., Jain, D., Hodonsky, C.J., Morrison, J.V., Davis, J.P., Brown, L., Sofer, T., Conomos, M.P., Schurmann, C., McHugh, C.P., et al. (2016). Genome-wide Association Study of Platelet Count Identifies Ancestry-Specific Loci in Hispanic/Latino Americans. Am. J. Hum. Genet. *98*, 229–242. https://doi.org/10.1016/j.ajhg.2015.12.003.
43. Hodonsky, C.J., Jain, D., Schick, U.M., Morrison, J.V., Brown, L., McHugh, C.P., Schurmann, C., Chen, D.D., Liu, Y.M., Auer, P.L., et al. (2017). Genome-wide association study of red blood cell traits in Hispanics/Latinos: The Hispanic Community Health Study/Study of Latinos. PLoS Genet. *13*, e1006760. https://doi.org/10.1371/journal.pgen.1006760.
44. Jain, D., Hodonsky, C.J., Schick, U.M., Morrison, J.V., Minnerath, S., Brown, L., Schurmann, C., Liu, Y., Auer, P. L., Laurie, C.A., et al. (2017). Genome-wide association of white blood cell counts in Hispanic/Latino Americans: the Hispanic Community Health Study/Study of Latinos. Hum. Mol. Genet. *26*, 1193–1204. https://doi.org/10.1093/hmg/ddx024.
45. Raffield, L.M., Louie, T., Sofer, T., Jain, D., Ipp, E., Taylor, K.D., Papanicolaou, G.J., Avilés-Santa, L., Lange, L.A., Laurie, C.C., et al. (2017). Genome-wide association study of iron traits and relation to diabetes in the Hispanic Community Health Study/Study of Latinos (HCHS/SOL): potential genomic intersection of iron and glucose regulation? Hum. Mol. Genet. *26*, 1966–1978. https://doi.org/10.1093/hmg/ddx082.
46. Sofer, T., Wong, Q., Hartwig, F.P., Taylor, K., Warren, H.R., Evangelou, E., Cabrera, C.P., Levy, D., Kramer, H., Lange, L.A., et al. (2017). Genome-Wide Association Study of Blood Pressure Traits by Hispanic/Latino Background: the Hispanic Community Health Study/Study of Latinos. Sci. Rep. *7*, 10348.
47. Jian, X., Sofer, T., Tarraf, W., Bressler, J., Faul, J.D., Zhao, W., Ratliff, S.M., Lamar, M., Launer, L.J., Laurie, C.C., et al. (2020). Genome-wide association study of cognitive function in diverse Hispanics/Latinos: results from the Hispanic

Community Health Study/Study of Latinos. Transl. Psychiatry *10*, 245.
48. Moon, J.Y., Louie, T.L., Jain, D., Sofer, T., Schurmann, C., Below, J.E., Lai, C.Q., Aviles-Santa, M.L., Talavera, G.A., Smith, C.E., et al. (2019). A Genome-Wide Association Study Identifies Blood Disorder-Related Variants Influencing Hemoglobin A_{1c} With Implications for Glycemic Status in U.S. Hispanics/Latinos. Diabetes Care *42*, 1784–1791. https://doi.org/10.2337/dc19-0168.
49. Graff, M., Emery, L.S., Justice, A.E., Parra, E., Below, J.E., Palmer, N.D., Gao, C., Duan, Q., Valladares-Salgado, A., Cruz, M., et al. (2017). Genetic architecture of lipid traits in the Hispanic community health study/study of Latinos. Lipids Health Dis. *16*, 200. https://doi.org/10.1186/s12944-017-0591-6.
50. Sofer, T., Emery, L., Jain, D., Ellis, A.M., Laurie, C.C., Allison, M.A., Lee, J., Kurniansyah, N., Kerr, K.F., González, H. M., et al. (2019). Variants Associated with the Ankle Brachial Index Differ by Hispanic/Latino Ethnic Group: a genome-wide association study in the Hispanic Community Health Study/Study of Latinos. Sci. Rep. *9*, 11410. https://doi.org/10.1038/s41598-019-47928-5.
51. Saccone, N.L., Emery, L.S., Sofer, T., Gogarten, S.M., Becker, D.M., Bottinger, E.P., Chen, L.S., Culverhouse, R.C., Duan, W., Hancock, D.B., et al. (2018). Genome-Wide Association Study of Heavy Smoking and Daily/Nondaily Smoking in the Hispanic Community Health Study/Study of Latinos (HCHS/SOL). Nicotine Tob. Res. *20*, 448–457. https://doi.org/10.1093/ntr/ntx107.
52. Burkart, K.M., Sofer, T., London, S.J., Manichaikul, A., Hartwig, F.P., Yan, Q., Soler Artigas, M., Avila, L., Chen, W., Davis Thomas, S., et al. (2018). A Genome-Wide Association Study in Hispanics/Latinos Identifies Novel Signals for Lung Function. The Hispanic Community Health Study/Study of Latinos. Am. J. Respir. Crit. Care Med. *198*, 208–219.
53. Dunn, E.C., Sofer, T., Gallo, L.C., Gogarten, S.M., Kerr, K.F., Chen, C.Y., Stein, M.B., Ursano, R.J., Guo, X., Jia, Y., et al. (2017). Genome-wide association study of generalized anxiety symptoms in the Hispanic Community Health Study/Study of Latinos. Am. J. Med. Genet. B Neuropsychiatr. *174*, 132–143. https://doi.org/10.1002/ajmg.b.32448.
54. Seyerle, A.A., Lin, H.J., Gogarten, S.M., Stilp, A., Méndez Giráldez, R., Soliman, E., Baldassari, A., Graff, M., Heckbert, S., Kerr, K.F., et al. (2018). Genome-wide association study of PR interval in Hispanics/Latinos identifies novel locus at ID2. Heart *104*, 904–911.
55. Qian, H., Kowalski, M.H., Kramer, H.J., Tao, R., Lash, J.P., Stilp, A.M., Cai, J., Li, Y., and Franceschini, N. (2020). Genome-Wide Association of Kidney Traits in Hispanics/Latinos Using Dense Imputed Whole-Genome Sequencing Data: The Hispanic Community Health Study/Study of Latinos. Circ. Genom. Precis. *13*, e002891. https://doi.org/10.1161/CIRCGEN.119.002891.
56. Feofanova, E.V., Chen, H., Dai, Y., Jia, P., Grove, M.L., Morrison, A.C., Qi, Q., Daviglus, M., Cai, J., North, K.E., et al. (2020). A Genome-wide Association Study Discovers 46 Loci of the Human Metabolome in the Hispanic Community Health Study/Study of Latinos. Am. J. Hum. Genet. *107*, 849–863.
57. Kurilshikov, A., Medina-Gomez, C., Bacigalupe, R., Radjabzadeh, D., Wang, J., Demirkan, A., Le Roy, C.I., Raygoza Garay, J.A., Finnicum, C.T., Liu, X., et al. (2021). Large-scale association analyses identify host factors influencing human gut microbiome composition. Nat. Genet. *53*, 156–165. https://doi.org/10.1038/s41588-020-00763-1.
58. Shungin, D., Haworth, S., Divaris, K., Agler, C.S., Kamatani, Y., Keun Lee, M., Grinde, K., Hindy, G., Alaraudanjoki, V., Pesonen, P., et al. (2019). Genome-wide analysis of dental caries and periodontitis combining clinical and self-reported data. Nat. Commun. *10*, 2773. https://doi.org/10.1038/s41467-019-10630-1.
59. Hu, Y., Bien, S.A., Nishimura, K.K., Haessler, J., Hodonsky, C.J., Baldassari, A.R., Highland, H.M., Wang, Z., Preuss, M., Sitlani, C. M., et al. (2021). Multi-ethnic genome-wide association analyses of white blood cell and platelet traits in the Population Architecture using Genomics and Epidemiology (PAGE) study. BMC Genomics *22*, 432. https://doi.org/10.1186/s12864-021-07745-5.
60. Liang, J., Le, T.H., Edwards, D.R.V., Tayo, B.O., Gaulton, K.J., Smith, J.A., Lu, Y., Jensen, R.A., Chen, G., Yanek, L.R., et al. (2017). Single-trait and multi-trait genome-wide association analyses identify novel loci for blood pressure in African-ancestry populations. PLoS Genet. *13*, e1006728. https://doi.org/10.1371/journal.pgen.1006728.
61. Sung, Y.J., Winkler, T.W., de las Fuentes, L., Bentley, A.R., Brown, M.R., Kraja, A.T., Schwander, K., Ntalla, I., Guo, X., Franceschini, N., et al. (2018). A Large-Scale Multi-ancestry Genome-wide Study Accounting for Smoking Behavior Identifies Multiple Significant Loci for Blood Pressure. Am. J. Hum. Genet. *102*, 375–400. https://doi.org/10.1016/j.ajhg.2018.01.015.
62. Yan, Q., Brehm, J., Pino-Yanes, M., Forno, E., Lin, J., Oh, S.S., Acosta-Perez, E., Laurie, C.C., Cloutier, M.M., Raby, B.A., et al. (2017). A meta-analysis of genome-wide association studies of asthma in Puerto Ricans. Eur. Respir. *49*, 1601505. https://doi.org/10.1183/13993003.01505-2016.
63. Zhao, X., Qiao, D., Yang, C., Kasela, S., Kim, W., Ma, Y., Shrine, N., Batini, C., Sofer, T., Taliun, S.A.G., et al. (2020). Whole genome sequence analysis of pulmonary function and COPD in 19,996 multi-ethnic participants. Nat. Commun. *11*, 5182. https://doi.org/10.1038/s41467-020-18334-7.
64. Kerr, K.F., Avery, C.L., Lin, H.J., Raffield, L.M., Zhang, Q.S., Browning, B.L., Browning, S.R., Conomos, M.P., Gogarten, S.M., Laurie, C.C., et al. (2017). Genome-wide association study of heart rate and its variability in Hispanic/Latino cohorts. Heart Rhythm *14*, 1675–1684. https://doi.org/10.1016/j.hrthm.2017.06.018.
65. Khoury, S., Wang, Q.P., Parisien, M., Gris, P., Bortsov, A.V., Linnstaedt, S.D., McLean, S.A., Tungate, A.S., Sofer, T., Lee, J., et al. (2021). Multi-ethnic GWAS and meta-analysis of sleep quality identify MPP6 as a novel gene that functions in sleep center neurons. Sleep *44*, zsaa211. https://doi.org/10.1093/sleep/zsaa211.
66. Méndez-Giráldez, R., Gogarten, S.M., Below, J.E., Yao, J., Seyerle, A.A., Highland, H.M., Kooperberg, C., Soliman, E.Z., Rotter, J.I., Kerr, K.F., et al. (2017). GWAS of the electrocardiographic QT interval in Hispanics/Latinos generalizes previously identified loci and identifies population-specific signals. Sci. Rep. *7*, 17075. https://doi.org/10.1038/s41598-017-17136-0.
67. Napier, M.D., Franceschini, N., Gondalia, R., Stewart, J.D., Méndez-Giráldez, R., Sitlani, C.M., Seyerle, A.A., Highland, H.M., Li, Y., Wilhelmsen, K.C., et al. (2018). Genome-wide

association study and meta-analysis identify loci associated with ventricular and supraventricular ectopy. Sci. Rep. *8*, 5675. https://doi.org/10.1038/s41598-018-23843-z.
68. Andaleon, A., Mogil, L.S., and Wheeler, H.E. (2019). Genetically regulated gene expression underlies lipid traits in Hispanic cohorts. PLoS One *14*, e0220827.
69. Horimoto, A.R.V.R., Xue, D., Cai, J., Lash, J.P., Daviglus, M.L., Franceschini, N., and Thornton, T.A. (2022). Genome-Wide Admixture Mapping of Estimated Glomerular Filtration Rate and Chronic Kidney Disease Identifies European and African Ancestry-of-Origin Loci in Hispanic and Latino Individuals in the United States. J. Am. Soc. Nephrol. *33*, 77–87.
70. Reynolds, K.M., Horimoto, A.R.V.R., Lin, B.M., Zhang, Y., Kurniansyah, N., Yu, B., Boerwinkle, E., Qi, Q., Kaplan, R., Daviglus, M., et al. (2023). Ancestry-driven metabolite variation provides insights into disease states in admixed populations. Genome Med. *15*, 52.
71. Hughes, E., Tshiaba, P., Gallagher, S., Wagner, S., Judkins, T., Roa, B., Rosenthal, E., Domchek, S., Garber, J., Lancaster, J., et al. (2020). Development and Validation of a Clinical Polygenic Risk Score to Predict Breast Cancer Risk. JCO Precis. Oncol. *4*, PO.19.00360.
72. O'Sullivan, J.W., Raghavan, S., Marquez-Luna, C., Luzum, J.A., Damrauer, S.M., Ashley, E.A., O'Donnell, C.J., Willer, C.J., Natarajan, P.; and American Heart Association Council on Genomic and Precision Medicine; Council on Clinical Cardiology; Council on Arteriosclerosis, Thrombosis and Vascular Biology; Council on Cardiovascular Radiology and Intervention; Council on Lifestyle and Cardiometabolic Health; and Council on Peripheral Vascular Disease (2022). Polygenic Risk Scores for Cardiovascular Disease: A Scientific Statement From the American Heart Association. Circulation *146*, e93–e118.
73. Kember, R.L., Merikangas, A.K., Verma, S.S., Verma, A., Judy, R., Regeneron Genetics Center, Damrauer, S.M., Ritchie, M.D., Rader, D.J., and Bućan, M. (2021). Polygenic Risk of Psychiatric Disorders Exhibits Cross-trait Associations in Electronic Health Record Data From European Ancestry Individuals. Biol. Psychiatry *89*, 236–245.
74. Guo, Y., Moon, J.Y., Laurie, C.C., North, K.E., Sanchez-Johnsen, L.A.P., Davis, S., Yu, B., Nyenhuis, S.M., Kaplan, R., Rastogi, D., and Qi, Q. (2018). Genetic predisposition to obesity is associated with asthma in US Hispanics/Latinos: Results from the Hispanic Community Health Study/Study of Latinos. Allergy *73*, 1547–1550.
75. Moon, J.Y., Wang, T., Sofer, T., North, K.E., Isasi, C.R., Cai, J., Gellman, M.D., Moncrieft, A.E., Sotres-Alvarez, D., Argos, M., et al. (2017). Objectively Measured Physical Activity, Sedentary Behavior, and Genetic Predisposition to Obesity in U.S. Hispanics/Latinos: Results From the Hispanic Community Health Study/Study of Latinos (HCHS/SOL). Diabetes *66*, 3001–3012.
76. Sofer, T., Moon, J.Y., Isasi, C.R., Qi, Q., Shah, N.A., Kaplan, R.C., and Kuniholm, M.H. (2018). Relationship of genetic determinants of height with cardiometabolic and pulmonary traits in the Hispanic Community Health Study/Study of Latinos. Int. J. Epidemiol. *47*, 2059–2069.
77. Preudhomme, L.K., Gellman, M.D., Franceschini, N., Perreira, K.M., Fernández-Rhodes, L.E., Gallo, L.C., Isasi, C.R., Smoller, S., Castañeda, S.F., Daviglus, M., et al. (2022). Genetic and stress influences on the prevalence of hypertension among hispanics/latinos in the hispanic community health study/study of latinos (HCHS/SOL). Blood Press. *31*, 155–163.
78. Zhang, Y., Elgart, M., Granot-Hershkovitz, E., Wang, H., Tarraf, W., Ramos, A.R., Stickel, A.M., Zeng, D., Garcia, T.P., Testai, F.D., et al. (2023). Genetic associations between sleep traits and cognitive ageing outcomes in the Hispanic Community Health Study/Study of Latinos. EBioMedicine *87*, 104393.
79. Fernandez-Rhodes, L., McArdle, C.E., Rao, H., Wang, Y., Martinez-Miller, E.E., Ward, J.B., Cai, J., Sofer, T., Isasi, C.R., and North, K.E. (2023). A Gene-Acculturation Study of Obesity Among US Hispanic/Latinos: The Hispanic Community Health Study/Study of Latinos. Psychosom. Med. *85*, 358–365.
80. Isasi, C.R., Moon, J.Y., Gallo, L.C., Qi, Q., Wang, T., Sotres-Alvarez, D., Llabre, M.M., Khambaty, T., Daviglus, M., Estrella, M.L., et al. (2022). Chronic Stress, Genetic Risk, and Obesity in US Hispanic/Latinos: Results From the Hispanic Community Health Study/Study of Latinos. Psychosom. Med. *84*, 822–827.
81. Privé, F., Arbel, J., and Vilhjálmsson, B.J. (2021). LDpred2: better, faster, stronger. Bioinformatics *36*, 5424–5431.
82. Zhang, Y., Elgart, M., Kurniansyah, N., Spitzer, B.W., Wang, H., Kim, D., Shah, N., Daviglus, M., Zee, P.C., Cai, J., et al. (2022). Genetic determinants of cardiometabolic and pulmonary phenotypes and obstructive sleep apnoea in HCHS/SOL. EBioMedicine *84*, 104288.
83. McArdle, C.E., Bokhari, H., Rodell, C.C., Buchanan, V., Preudhomme, L.K., Isasi, C.R., Graff, M., North, K., Gallo, L.C., Pirzada, A., et al. (2021). Findings from the Hispanic Community Health Study/Study of Latinos on the Importance of Sociocultural Environmental Interactors: Polygenic Risk Score-by-Immigration and Dietary Interactions. Front. Genet. *12*, 720750.
84. Ge, T., Chen, C.Y., Ni, Y., Feng, Y.C.A., and Smoller, J.W. (2019). Polygenic prediction via Bayesian regression and continuous shrinkage priors. Nat. Commun. *10*, 1776.
85. Zhou, L.Y., Sofer, T., Horimoto, A.R.V.R., Talavera, G.A., Lash, J.P., Cai, J., and Franceschini, N. (2023). Polygenic risk scores and kidney traits in the Hispanic/Latino population: The Hispanic Community Health Study/Study of Latinos. HGG Adv. *4*, 100177.
86. Kurniansyah, N., Goodman, M.O., Kelly, T.N., Elfassy, T., Wiggins, K.L., Bis, J.C., Guo, X., Palmas, W., Taylor, K.D., Lin, H.J., et al. (2022). A multi-ethnic polygenic risk score is associated with hypertension prevalence and progression throughout adulthood. Nat. Commun. *13*, 3549.
87. Kurniansyah, N., Goodman, M.O., Khan, A.T., Wang, J., Feofanova, E., Bis, J.C., Wiggins, K.L., Huffman, J.E., Kelly, T., Elfassy, T., et al. (2023). Evaluating the use of blood pressure polygenic risk scores across race/ethnic background groups. Nat. Commun. *14*, 3202.
88. Sofer, T., Kurniansyah, N., Granot-Hershkovitz, E., Goodman, M.O., Tarraf, W., Broce, I., Lipton, R.B., Daviglus, M., Lamar, M., Wassertheil-Smoller, S., et al. (2023). A polygenic risk score for Alzheimer's disease constructed using APOE-region variants has stronger association than APOE alleles with mild cognitive impairment in Hispanic/Latino adults in the U.S. Alzheimers Res. Ther. *15*, 146.
89. Grinde, K.E., Qi, Q., Thornton, T.A., Liu, S., Shadyab, A.H., Chan, K.H.K., Reiner, A.P., and Sofer, T. (2019). Generalizing polygenic risk scores from Europeans to Hispanics/Latinos. Genet. Epidemiol. *43*, 50–62.
90. Spear, M.L., Diaz-Papkovich, A., Ziv, E., Yracheta, J.M., Gravel, S., Torgerson, D.G., and Hernandez, R.D. (2020).

Recent shifts in the genomic ancestry of Mexican Americans may alter the genetic architecture of biomedical traits. Elife *9*, e56029.
91. Fernández-Rhodes, L., McArdle, C.E., Rao, H., Wang, Y., Martinez-Miller, E.E., Ward, J.B., Cai, J., Sofer, T., Isasi, C.R., and North, K.E. (2023). A Gene-Acculturation Study of Obesity Among US Hispanic/Latinos: The Hispanic Community Health Study/Study of Latinos. Psychosom. Med. *85*, 358–365.
92. Hu, Y., Graff, M., Haessler, J., Buyske, S., Bien, S.A., Tao, R., Highland, H.M., Nishimura, K.K., Zubair, N., Lu, Y., et al. (2020). Minority-centric meta-analyses of blood lipid levels identify novel loci in the Population Architecture using Genomics and Epidemiology (PAGE) study. PLoS Genet. *16*, e1008684.
93. Hu, X., Qiao, D., Kim, W., Moll, M., Balte, P.P., Lange, L.A., Bartz, T.M., Kumar, R., Li, X., Yu, B., et al. (2022). Polygenic transcriptome risk scores for COPD and lung function improve cross-ethnic portability of prediction in the NHLBI TOPMed program. Am. J. Hum. Genet. *109*, 857–870.
94. Anwar, M.Y., Graff, M., Highland, H.M., Smit, R., Wang, Z., Buchanan, V.L., Young, K.L., Kenny, E.E., Fernandez-Rhodes, L., Liu, S., et al. (2023). Assessing efficiency of fine-mapping obesity-associated variants through leveraging ancestry architecture and functional annotation using PAGE and UKBB cohorts. Hum. Genet. *142*, 1477–1489.
95. Downie, C.G., Dimos, S.F., Bien, S.A., Hu, Y., Darst, B.F., Polfus, L.M., Wang, Y., Wojcik, G.L., Tao, R., Raffield, L.M., et al. (2022). Multi-ethnic GWAS and fine-mapping of glycaemic traits identify novel loci in the PAGE Study. Diabetologia *65*, 477–489.
96. Fernandez-Rhodes, L., Gong, J., Haessler, J., Franceschini, N., Graff, M., Nishimura, K.K., Wang, Y., Highland, H.M., Yoneyama, S., Bush, W.S., et al. (2017). Trans-ethnic fine-mapping of genetic loci for body mass index in the diverse ancestral populations of the Population Architecture using Genomics and Epidemiology (PAGE) Study reveals evidence for multiple signals at established loci. Hum. Genet. *136*, 771–800.
97. Franceschini, N., Carty, C.L., Lu, Y., Tao, R., Sung, Y.J., Manichaikul, A., Haessler, J., Fornage, M., Schwander, K., Zubair, N., et al. (2016). Variant Discovery and Fine Mapping of Genetic Loci Associated with Blood Pressure Traits in Hispanics and African Americans. PLoS One *11*, e0164132.
98. Zubair, N., Graff, M., Luis Ambite, J., Bush, W.S., Kichaev, G., Lu, Y., Manichaikul, A., Sheu, W.H.H., Absher, D., Assimes, T.L., et al. (2016). Fine-mapping of lipid regions in global populations discovers ethnic-specific signals and refines previously identified lipid loci. Hum. Mol. Genet. *25*, 5500–5512.
99. Fernandez-Rhodes, L., Graff, M., Buchanan, V.L., Justice, A.E., Highland, H.M., Guo, X., Zhu, W., Chen, H.H., Young, K.L., Adhikari, K., et al. (2022). Ancestral diversity improves discovery and fine-mapping of genetic loci for anthropometric traits-The Hispanic/Latino Anthropometry Consortium. HGG Adv *3*, 100099.
100. Kocarnik, J.M., Richard, M., Graff, M., Haessler, J., Bien, S., Carlson, C., Carty, C.L., Reiner, A.P., Avery, C.L., Ballantyne, C.M., et al. (2018). Discovery, fine-mapping, and conditional analyses of genetic variants associated with C-reactive protein in multiethnic populations using the Metabochip in the Population Architecture using Genomics and Epidemiology (PAGE) study. Hum. Mol. Genet. *27*, 2940–2953.
101. Jo Hodonsky, C., Schurmann, C., Schick, U.M., Kocarnik, J., Tao, R., van Rooij, F.J., Wassel, C., Buyske, S., Fornage, M., Hindorff, L.A., et al. (2018). Generalization and fine mapping of red blood cell trait genetic associations to multi-ethnic populations: The PAGE Study. Am. J. Hematol. *93*, 1061–1073.
102. Fernandez-Rhodes, L., Malinowski, J.R., Wang, Y., Tao, R., Pankratz, N., Jeff, J.M., Yoneyama, S., Carty, C. L., Setiawan, V.W., Marchand, L.L., et al. (2018). The genetic underpinnings of variation in ages at menarche and natural menopause among women from the multi-ethnic Population Architecture using Genomics and Epidemiology (PAGE) Study: A trans-ethnic meta-analysis. PLoS One *13*, e0200486.
103. Wojcik, G.L., Graff, M., Nishimura, K.K., Tao, R., Haessler, J., Gignoux, C.R., Highland, H.M., Patel, Y.M., Sorokin, E.P., Avery, C.L., et al. (2019). Genetic analyses of diverse populations improves discovery for complex traits. Nature *570*, 514–518.
104. Bien, S.A., Pankow, J.S., Haessler, J., Lu, Y., Pankratz, N., Rohde, R.R., Tamuno, A., Carlson, C.S., Schumacher, F.R., Bůžková, P., et al. (2017). Transethnic insight into the genetics of glycaemic traits: fine-mapping results from the Population Architecture using Genomics and Epidemiology (PAGE) consortium. Diabetologia *60*, 2384–2398.
105. Burgess, S., Davey Smith, G., Davies, N.M., Dudbridge, F., Gill, D., Glymour, M.M., Hartwig, F.P., Kutalik, Z., Holmes, M.V., Minelli, C., et al. (2019). Guidelines for performing Mendelian randomization investigations: update for summer 2023. Wellcome Open Res. *4*, 186.
106. Scannell Bryan, M., Sofer, T., Mossavar-Rahmani, Y., Thyagarajan, B., Zeng, D., Daviglus, M.L., and Argos, M. (2019). Mendelian randomization of inorganic arsenic metabolism as a risk factor for hypertension- and diabetes-related traits among adults in the Hispanic Community Health Study/Study of Latinos (HCHS/SOL) cohort. Int. J. Epidemiol. *48*, 876–886.
107. Scannell Bryan, M., Sofer, T., Afshar, M., Mossavar-Rahmani, Y., Hosgood, H.D., Punjabi, N.M., Zeng, D., Daviglus, M.L., and Argos, M. (2021). Mendelian randomization analysis of arsenic metabolism and pulmonary function within the Hispanic Community Health Study/Study of Latinos. Sci. Rep. *11*, 13470.
108. Lee, Y., Chen, H., Chen, W., Qi, Q., Afshar, M., Cai, J., Daviglus, M.L., Thyagarajan, B., North, K.E., London, S.J., et al. (2022). Metabolomic Associations of Asthma in the Hispanic Community Health Study/Study of Latinos. Metabolites *12*, 359.
109. Granot-Hershkovitz, E., He, S., Bressler, J., Yu, B., Tarraf, W., Rebholz, C.M., Cai, J., Chan, Q., Garcia, T.P., Mosley, T., et al. (2023). Plasma metabolites associated with cognitive function across race/ethnicities affirming the importance of healthy nutrition. Alzheimers Dement. *19*, 1331–1342.
110. Bentley, A.R., Sung, Y.J., Brown, M.R., Winkler, T.W., Kraja, A.T., Ntalla, I., Schwander, K., Chasman, D.I., Lim, E., Deng, X., et al. (2019). Multi-ancestry genome-wide gene-smoking interaction study of 387,272 individuals identifies new loci associated with serum lipids. Nat. Genet. *51*, 636–648.
111. de Las Fuentes, L., Sung, Y.J., Noordam, R., Winkler, T., Feitosa, M.F., Schwander, K., Bentley, A.R., Brown, M.R., Guo, X., Manning, A., et al. (2021). Gene-educational attainment interactions in a multi-ancestry genome-wide meta-analysis

identify novel blood pressure loci. Mol. Psychiatry *26*, 2111–2125.
112. de Vries, P.S., Brown, M.R., Bentley, A.R., Sung, Y.J., Winkler, T.W., Ntalla, I., Schwander, K., Kraja, A.T., Guo, X., Franceschini, N., et al. (2019). Multiancestry Genome-Wide Association Study of Lipid Levels Incorporating Gene-Alcohol Interactions. Am. J. Epidemiol. *188*, 1033–1054.
113. Loos, R.J.F., and Yeo, G.S.H. (2022). The genetics of obesity: from discovery to biology. Nat. Rev. Genet. *23*, 120–133.
114. Noordam, R., Sitlani, C.M., Avery, C.L., Stewart, J.D., Gogarten, S.M., Wiggins, K.L., Trompet, S., Warren, H.R., Sun, F., Evans, D.S., et al. (2017). A genome-wide interaction analysis of tricyclic/tetracyclic antidepressants and RR and QT intervals: a pharmacogenomics study from the Cohorts for Heart and Aging Research in Genomic Epidemiology (CHARGE) consortium. J. Med. Genet. *54*, 313–323.
115. Melin, K., Moon, J.Y., Qi, Q., Hernandez-Suarez, D.F., Duconge, J., Hua, S., Gonzalez, S., Zeng, D., and Kaplan, R.C. (2019). Prevalence of pharmacogenomic variants affecting the efficacy of clopidogrel therapy in the Hispanic Community Health Study/Study of Latinos cohort. Pharmacogenomics *20*, 75–83.
116. Han, X., Lains, I., Li, J., Li, J., Chen, Y., Yu, B., Qi, Q., Boerwinkle, E., Kaplan, R., Thyagarajan, B., et al. (2023). Integrating genetics and metabolomics from multi-ethnic and multi-fluid data reveals putative mechanisms for age-related macular degeneration. Cell Rep. Med. *4*, 101085.
117. Luo, K., Chen, G.C., Zhang, Y., Moon, J.Y., Xing, J., Peters, B.A., Usyk, M., Wang, Z., Hu, G., Li, J., et al. (2024). Variant of the lactase LCT gene explains association between milk intake and incident type 2 diabetes. Nat. Metab. *6*, 169–186.
118. Qi, Q., Li, J., Yu, B., Moon, J.Y., Chai, J.C., Merino, J., Hu, J., Ruiz-Canela, M., Rebholz, C., Wang, Z., et al. (2022). Host and gut microbial tryptophan metabolism and type 2 diabetes: an integrative analysis of host genetics, diet, gut microbiome and circulating metabolites in cohort studies. Gut *71*, 1095–1105.
119. Gieger, C., Geistlinger, L., Altmaier, E., Hrabé de Angelis, M., Kronenberg, F., Meitinger, T., Mewes, H.W., Wichmann, H.E., Weinberger, K.M., Adamski, J., et al. (2008). Genetics meets metabolomics: a genome-wide association study of metabolite profiles in human serum. PLoS Genet. *4*, e1000282.
120. Yang, C., Hallmark, B., Chai, J.C., O'Connor, T.D., Reynolds, L.M., Wood, A.C., Seeds, M., Chen, Y.D.I., Steffen, L.M., Tsai, M.Y., et al. (2021). Impact of Amerind ancestry and FADS genetic variation on omega-3 deficiency and cardiometabolic traits in Hispanic populations. Commun. Biol. *4*, 918.
121. Kurniansyah, N., Wallace, D.A., Zhang, Y., Yu, B., Cade, B., Wang, H., Ochs-Balcom, H.M., Reiner, A.P., Ramos, A.R., Smith, J.D., et al. (2023). An integrated multi-omics analysis of sleep-disordered breathing traits implicates P2XR4 purinergic signaling. Commun. Biol. *6*, 125.
122. Nelson, S.C., Gogarten, S.M., Fullerton, S.M., Isasi, C.R., Mitchell, B.D., North, K.E., Rich, S.S., Taylor, M.R.G., Zöllner, S., and Sofer, T. (2022). Social and scientific motivations to move beyond groups in allele frequencies: The TOPMed experience. Am. J. Hum. Genet. *109*, 1582–1590.
123. Gonzalez, S., Strizich, G., Isasi, C.R., Hua, S., Comas, B., Sofer, T., Thyagarajan, B., Perreira, K.M., Talavera, G.A., Daviglus, M.L., et al. (2021). Consent for Use of Genetic Data among US Hispanics/Latinos: Results from the Hispanic Community Health Study/Study of Latinos. Ethn. Dis. *31*, 547–558.
124. Christensen, K.D., Zhang, M., Galbraith, L.N., Granot-Hershkovitz, E., Nelson, S.C., Gonzalez, S., Argos, M., Perreira, K.M., Daviglus, M.L., Isasi, C.R., et al. (2023). Awareness and utilization of genetic testing among Hispanic and Latino adults living in the US: The Hispanic Community Health Study/Study of Latinos. HGG Adv. *4*, 100160.
125. (2023). Using Population Descriptors in Genetics and Genomics Research: A New Framework for an Evolving Field (The National Academies Collection: Reports funded by National Institutes of Health), pp. 29–31.
126. Sankar, P., and Cho, M.K. (2002). Genetics. Toward a new vocabulary of human genetic variation. Science *298*, 1337–1338.
127. Bonham, V.L., Green, E.D., and Pérez-Stable, E.J. (2018). Examining How Race, Ethnicity, and Ancestry Data Are Used in Biomedical Research. JAMA *320*, 1533–1534.
128. Khan, A.T., Gogarten, S.M., McHugh, C.P., Stilp, A.M., Sofer, T., Bowers, M.L., Wong, Q., Cupples, L.A., Hidalgo, B., Johnson, A.D., et al. (2022). Recommendations on the use and reporting of race, ethnicity, and ancestry in genetic research: Experiences from the NHLBI TOPMed program. Cell Genom. *2*, 100155.
129. Browning, S.R., Grinde, K., Plantinga, A., Gogarten, S.M., Stilp, A.M., Kaplan, R.C., Avilés-Santa, M.L., Browning, B.L., and Laurie, C.C. (2016). Local Ancestry Inference in a Large US-Based Hispanic/Latino Study: Hispanic Community Health Study/Study of Latinos (HCHS/SOL). G3 (Bethesda) *6*, 1525–1534.
130. Kumuthini, J., Zick, B., Balasopoulou, A., Chalikiopoulou, C., Dandara, C., El-Kamah, G., Findley, L., Katsila, T., Li, R., Maceda, E.B., et al. (2022). The clinical utility of polygenic risk scores in genomic medicine practices: a systematic review. Hum. Genet. *141*, 1697–1704.
131. Slunecka, J.L., van der Zee, M.D., Beck, J.J., Johnson, B.N., Finnicum, C.T., Pool, R., Hottenga, J.J., de Geus, E.J.C., and Ehli, E.A. (2021). Implementation and implications for polygenic risk scores in healthcare. Hum. Genomics *15*, 46.
132. Legge, S.E., Pardiñas, A.F., Helthuis, M., Jansen, J.A., Jollie, K., Knapper, S., MacCabe, J.H., Rujescu, D., Collier, D.A., O'Donovan, M.C., et al. (2019). A genome-wide association study in individuals of African ancestry reveals the importance of the Duffy-null genotype in the assessment of clozapine-related neutropenia. Mol. Psychiatry *24*, 328–337.
133. Joshi, P.H., Marcovina, S., Orroth, K., López, J.A.G., Kent, S.T., Kaplan, R., Swett, K., Sotres-Alvarez, D., Thyagarajan, B., Slipczuk, L., et al. (2023). Heterogeneity of Lipoprotein(a) Levels Among Hispanic or Latino Individuals Residing in the US. JAMA Cardiol. *8*, 691–696.
134. Pinheiro, P.S., Callahan, K.E., and Kobetz, E.N. (2020). Disaggregated Hispanic Groups and Cancer: Importance, Methodology, and Current Knowledge. In Advancing the Science of Cancer in Latinos, A.G. Ramirez and E.J. Trapido, eds. (Cham (CH): Springer), pp. 17–34. Chapter 2. https://www.ncbi.nlm.nih.gov/books/NBK573253/.
135. Westerman, K.E., and Sofer, T. (2024). Many roads to a gene-environment interaction. Am. J. Hum. Genet. *111*, 626–635.
136. Kapoor, M., Chao, M.J., Johnson, E.C., Novikova, G., Lai, D., Meyers, J.L., Schulman, J., Nurnberger, J.I., Jr., Porjesz, B., Liu, Y., et al. (2021). Multi-omics integration analysis identifies novel genes for alcoholism with potential overlap with neurodegenerative diseases. Nat. Commun. *12*, 5071.

137. van Duijvenboden, S., Ramírez, J., Young, W.J., Olczak, K.J., Ahmed, F., Alhammadi, M.J.A.Y., International Consortium of Blood Pressure, Bell, C.G., Morris, A.P., and Munroe, P.B. (2023). Integration of genetic fine-mapping and multi-omics data reveals candidate effector genes for hypertension. Am. J. Hum. Genet. *110*, 1718–1734.
138. Liang, Y., Pividori, M., Manichaikul, A., Palmer, A.A., Cox, N.J., Wheeler, H.E., and Im, H.K. (2022). Polygenic transcriptome risk scores (PTRS) can improve portability of polygenic risk scores across ancestries. Genome Biol. *23*, 23.
139. Ambale-Venkatesh, B., Yang, X., Wu, C.O., Liu, K., Hundley, W.G., McClelland, R., Gomes, A.S., Folsom, A.R., Shea, S., Guallar, E., et al. (2017). Cardiovascular Event Prediction by Machine Learning: The Multi-Ethnic Study of Atherosclerosis. Circ. Res. *121*, 1092–1101.
140. Azodi, C.B., Bolger, E., McCarren, A., Roantree, M., de Los Campos, G., and Shiu, S.H. (2019). Benchmarking Parametric and Machine Learning Models for Genomic Prediction of Complex Traits. G3 (Bethesda). G3 (Bethesda). *9*, 3691–3702.
141. Moyon, L., Berthelot, C., Louis, A., Nguyen, N.T.T., and Roest Crollius, H. (2022). Classification of non-coding variants with high pathogenic impact. PLoS Genet. *18*, e1010191.
142. Shigemizu, D., Akiyama, S., Suganuma, M., Furutani, M., Yamakawa, A., Nakano, Y., Ozaki, K., and Niida, S. (2023). Classification and deep-learning-based prediction of Alzheimer disease subtypes by using genomic data. Transl. Psychiatry *13*, 232.
143. Roman-Naranjo, P., Parra-Perez, A.M., and Lopez-Escamez, J.A. (2023). A systematic review on machine learning approaches in the diagnosis and prognosis of rare genetic diseases. J. Biomed. Inform. *143*, 104429.
144. Azodi, C.B., Tang, J., and Shiu, S.H. (2020). Opening the Black Box: Interpretable Machine Learning for Geneticists. Trends Genet. *36*, 442–455.
145. Hrytsenko, Y., Shea, B., Elgart, M., Kurniansyah, N., Lyons, G., Morrison, A.C., Carson, A.P., Haring, B., Mitchell, B.D., Psaty, B.M., et al. (2024). Machine learning models for predicting blood pressure phenotypes by combining multiple polygenic risk scores. Sci. Rep. *14*, 12436.
146. Deng, L., Ruiz-Linares, A., Xu, S., and Wang, S. (2016). Ancestry variation and footprints of natural selection along the genome in Latin American populations. Sci. Rep. *6*, 21766.
147. Norris, E.T., Rishishwar, L., Chande, A.T., Conley, A.B., Ye, K., Valderrama-Aguirre, A., and Jordan, I.K. (2020). Admixture-enabled selection for rapid adaptive evolution in the Americas. Genome Biol. *21*, 29.
148. Fumagalli, M., Sironi, M., Pozzoli, U., Ferrer-Admetlla, A., Pattini, L., and Nielsen, R. (2011). Signatures of environmental genetic adaptation pinpoint pathogens as the main selective pressure through human evolution. PLoS Genet. *7*, e1002355.
149. Norris, E.T., Rishishwar, L., Wang, L., Conley, A.B., Chande, A.T., Dabrowski, A.M., Valderrama-Aguirre, A., and Jordan, I.K. (2019). Assortative Mating on Ancestry-Variant Traits in Admixed Latin American Populations. Front. Genet. *10*, 359.
150. Pollen, A.A., Kilik, U., Lowe, C.B., and Camp, J.G. (2023). Human-specific genetics: new tools to explore the molecular and cellular basis of human evolution. Nat. Rev. Genet. *24*, 687–711.
151. Goodman, M.O., Cade, B.E., Shah, N.A., Huang, T., Dashti, H.S., Saxena, R., Rutter, M.K., Libby, P., Sofer, T., and Redline, S. (2022). Pathway-Specific Polygenic Risk Scores Identify Obstructive Sleep Apnea-Related Pathways Differentially Moderating Genetic Susceptibility to Coronary Artery Disease. Circ. Genom. Precis. Med. *15*, e003535.
152. Elgart, M., Lyons, G., Romero-Brufau, S., Kurniansyah, N., Brody, J.A., Guo, X., Lin, H.J., Raffield, L., Gao, Y., Chen, H., et al. (2022). Non-linear machine learning models incorporating SNPs and PRS improve polygenic prediction in diverse human populations. Commun. Biol. *5*, 856.
153. Bagheri, M., Chung, C.P., Dickson, A.L., Van Driest, S.L., Borinstein, S.C., and Mosley, J.D. (2023). White blood cell ranges and frequency of neutropenia by Duffy genotype status. Blood Adv. *7*, 406–409.

HGGA, Volume *6*

Supplemental information

Advancements in genetic research by the Hispanic Community Health Study/Study of Latinos: A 10-year retrospective review

Hridya Rao, Margaret C. Weiss, Jee Young Moon, Krista M. Perreira, Martha L. Daviglus, Robert Kaplan, Kari E. North, Maria Argos, Lindsay Fernández-Rhodes, and Tamar Sofer

Supplementary Table

Table S1: Summary of genetic epidemiological studies for health outcomes in HCHS/SOL

Study type	Health outcome or trait
Genome wide association studies	Metabolome (1)<br>Blood traits (2-6)<br>Pulmonary disease and function (7-9)<br>Cardiometabolic traits (10-15)<br>Cardiovascular conduction (16-20)<br>Kidney disease and function (21)<br>Cognitive function, behavior, depression, and anxiety (22-26)<br>Dental conditions (27-30)
Admixture mapping	Cardiometabolic traits (31, 32)<br>Kidney traits (33, 34)<br>Pulmonary function (7, 35)
Polygenic risk score	Anthropometrics and obesity (36-41)<br>Cardiometabolic traits (37, 42-44)<br>Cognition and psychiatric traits (22, 23)<br>Pulmonary function (37, 42, 45, 46)
Fine mapping	Anthropometrics (47, 48)<br>Cardiometabolic traits (18, 43, 49-53)<br>Kidney function (54)<br>Reproductive traits (55)<br>Blood traits (56, 57)
Mendelian Randomization	Cardiometabolic traits (58)<br>Pulmonary function (42, 59, 60)<br>Cognition (61)
Gene by Environment Interaction	Anthropometrics and obesity (36, 38)<br>Cardiometabolic traits (62, 63)<br>Cognition and psychiatric traits (64)
Metabolomics integration	Cardiometabolic traits (1, 65-67)<br>Cognition and sleep (68, 69)<br>Macular Degeneration (70)

Supplementary references

1. Feofanova EV, Chen H, Dai Y, Jia P, Grove ML, Morrison AC, et al. A Genome-wide Association Study Discovers 46 Loci of the Human Metabolome in the Hispanic Community Health Study/Study of Latinos. Am J Hum Genet. 2020;107(5):849-63.
2. Moon JY, Louie TL, Jain D, Sofer T, Schurmann C, Below JE, et al. A Genome-Wide Association Study Identifies Blood Disorder-Related Variants Influencing Hemoglobin A(1c) With Implications for Glycemic Status in U.S. Hispanics/Latinos. Diabetes Care. 2019;42(9):1784-91.
3. Jain D, Hodonsky CJ, Schick UM, Morrison JV, Minnerath S, Brown L, et al. Genome-wide association of white blood cell counts in Hispanic/Latino Americans: the Hispanic Community Health Study/Study of Latinos. Hum Mol Genet. 2017;26(6):1193-204.
4. Raffield LM, Louie T, Sofer T, Jain D, Ipp E, Taylor KD, et al. Genome-wide association study of iron traits and relation to diabetes in the Hispanic Community Health Study/Study of Latinos (HCHS/SOL): potential genomic intersection of iron and glucose regulation? Hum Mol Genet. 2017;26(10):1966-78.
5. Schick UM, Jain D, Hodonsky CJ, Morrison JV, Davis JP, Brown L, et al. Genome-wide Association Study of Platelet Count Identifies Ancestry-Specific Loci in Hispanic/Latino Americans. Am J Hum Genet. 2016;98(2):229-42.
6. Hodonsky CJ, Jain D, Schick UM, Morrison JV, Brown L, McHugh CP, et al. Genome-wide association study of red blood cell traits in Hispanics/Latinos: The Hispanic Community Health Study/Study of Latinos. PLoS Genet. 2017;13(4):e1006760.
7. Burkart KM, Sofer T, London SJ, Manichaikul A, Hartwig FP, Yan Q, et al. A Genome-Wide Association Study in Hispanics/Latinos Identifies Novel Signals for Lung Function. The Hispanic Community Health Study/Study of Latinos. Am J Respir Crit Care Med. 2018;198(2):208-19.
8. Yan Q, Brehm J, Pino-Yanes M, Forno E, Lin J, Oh SS, et al. A meta-analysis of genome-wide association studies of asthma in Puerto Ricans. Eur Respir J. 2017;49(5).
9. Zhao X, Qiao D, Yang C, Kasela S, Kim W, Ma Y, et al. Whole genome sequence analysis of pulmonary function and COPD in 19,996 multi-ethnic participants. Nat Commun. 2020;11(1):5182.
10. Sofer T, Wong Q, Hartwig FP, Taylor K, Warren HR, Evangelou E, et al. Genome-Wide Association Study of Blood Pressure Traits by Hispanic/Latino Background: the Hispanic Community Health Study/Study of Latinos. Sci Rep. 2017;7(1):10348.
11. Justice AE, Young K, Gogarten SM, Sofer T, Graff M, Love SAM, et al. Genome-wide association study of body fat distribution traits in Hispanics/Latinos from the HCHS/SOL. Hum Mol Genet. 2021;30(22):2190-204.
12. Qi Q, Stilp AM, Sofer T, Moon JY, Hidalgo B, Szpiro AA, et al. Genetics of Type 2 Diabetes in U.S. Hispanic/Latino Individuals: Results From the Hispanic Community Health Study/Study of Latinos (HCHS/SOL). Diabetes. 2017;66(5):1419-25.
13. Sung YJ, Winkler TW, de Las Fuentes L, Bentley AR, Brown MR, Kraja AT, et al. A Large-Scale Multi-ancestry Genome-wide Study Accounting for Smoking Behavior Identifies Multiple Significant Loci for Blood Pressure. Am J Hum Genet. 2018;102(3):375-400.
14. Liang J, Le TH, Edwards DRV, Tayo BO, Gaulton KJ, Smith JA, et al. Single-trait and multi-trait genome-wide association analyses identify novel loci for blood pressure in African-ancestry populations. PLoS Genet. 2017;13(5):e1006728.
15. Sofer T, Emery L, Jain D, Ellis AM, Laurie CC, Allison MA, et al. Variants Associated with the Ankle Brachial Index Differ by Hispanic/Latino Ethnic Group: a genome-wide

association study in the Hispanic Community Health Study/Study of Latinos. Sci Rep. 2019;9(1):11410.
16. Napier MD, Franceschini N, Gondalia R, Stewart JD, Mendez-Giraldez R, Sitlani CM, et al. Genome-wide association study and meta-analysis identify loci associated with ventricular and supraventricular ectopy. Sci Rep. 2018;8(1):5675.
17. Kerr KF, Avery CL, Lin HJ, Raffield LM, Zhang QS, Browning BL, et al. Genome-wide association study of heart rate and its variability in Hispanic/Latino cohorts. Heart Rhythm. 2017;14(11):1675-84.
18. Seyerle AA, Lin HJ, Gogarten SM, Stilp A, Mendez Giraldez R, Soliman E, et al. Genome-wide association study of PR interval in Hispanics/Latinos identifies novel locus at ID2. Heart. 2018;104(11):904-11.
19. Swenson BR, Louie T, Lin HJ, Mendez-Giraldez R, Below JE, Laurie CC, et al. GWAS of QRS duration identifies new loci specific to Hispanic/Latino populations. PLoS One. 2019;14(6):e0217796.
20. Mendez-Giraldez R, Gogarten SM, Below JE, Yao J, Seyerle AA, Highland HM, et al. GWAS of the electrocardiographic QT interval in Hispanics/Latinos generalizes previously identified loci and identifies population-specific signals. Sci Rep. 2017;7(1):17075.
21. Qian H, Kowalski MH, Kramer HJ, Tao R, Lash JP, Stilp AM, et al. Genome-Wide Association of Kidney Traits in Hispanics/Latinos Using Dense Imputed Whole-Genome Sequencing Data: The Hispanic Community Health Study/Study of Latinos. Circ Genom Precis Med. 2020;13(4):e002891.
22. Jian X, Sofer T, Tarraf W, Bressler J, Faul JD, Zhao W, et al. Genome-wide association study of cognitive function in diverse Hispanics/Latinos: results from the Hispanic Community Health Study/Study of Latinos. Transl Psychiatry. 2020;10(1):245.
23. Dunn EC, Sofer T, Wang MJ, Soare TW, Gallo LC, Gogarten SM, et al. Genome-wide association study of depressive symptoms in the Hispanic Community Health Study/Study of Latinos. J Psychiatr Res. 2018;99:167-76.
24. Dunn EC, Sofer T, Gallo LC, Gogarten SM, Kerr KF, Chen CY, et al. Genome-wide association study of generalized anxiety symptoms in the Hispanic Community Health Study/Study of Latinos. Am J Med Genet B Neuropsychiatr Genet. 2017;174(2):132-43.
25. Saccone NL, Emery LS, Sofer T, Gogarten SM, Becker DM, Bottinger EP, et al. Genome-Wide Association Study of Heavy Smoking and Daily/Nondaily Smoking in the Hispanic Community Health Study/Study of Latinos (HCHS/SOL). Nicotine Tob Res. 2018;20(4):448-57.
26. Khoury S, Wang QP, Parisien M, Gris P, Bortsov AV, Linnstaedt SD, et al. Multi-ethnic GWAS and meta-analysis of sleep quality identify MPP6 as a novel gene that functions in sleep center neurons. Sleep. 2021;44(3).
27. Sanders AE, Sofer T, Wong Q, Kerr KF, Agler C, Shaffer JR, et al. Chronic Periodontitis Genome-wide Association Study in the Hispanic Community Health Study / Study of Latinos. J Dent Res. 2017;96(1):64-72.
28. Shungin D, Haworth S, Divaris K, Agler CS, Kamatani Y, Keun Lee M, et al. Genome-wide analysis of dental caries and periodontitis combining clinical and self-reported data. Nat Commun. 2019;10(1):2773.
29. Morrison J, Laurie CC, Marazita ML, Sanders AE, Offenbacher S, Salazar CR, et al. Genome-wide association study of dental caries in the Hispanic Communities Health Study/Study of Latinos (HCHS/SOL). Hum Mol Genet. 2016;25(4):807-16.
30. Sanders AE, Jain D, Sofer T, Kerr KF, Laurie CC, Shaffer JR, et al. GWAS Identifies New Loci for Painful Temporomandibular Disorder: Hispanic Community Health Study/Study of Latinos. J Dent Res. 2017;96(3):277-84.

31. Sofer T, Baier LJ, Browning SR, Thornton TA, Talavera GA, Wassertheil-Smoller S, et al. Admixture mapping in the Hispanic Community Health Study/Study of Latinos reveals regions of genetic associations with blood pressure traits. PLoS One. 2017;12(11):e0188400.
32. Andaleon A, Mogil LS, Wheeler HE. Genetically regulated gene expression underlies lipid traits in Hispanic cohorts. PLoS One. 2019;14(8):e0220827.
33. Brown LA, Sofer T, Stilp AM, Baier LJ, Kramer HJ, Masindova I, et al. Admixture Mapping Identifies an Amerindian Ancestry Locus Associated with Albuminuria in Hispanics in the United States. J Am Soc Nephrol. 2017;28(7):2211-20.
34. Horimoto A, Xue D, Cai J, Lash JP, Daviglus ML, Franceschini N, et al. Genome-Wide Admixture Mapping of Estimated Glomerular Filtration Rate and Chronic Kidney Disease Identifies European and African Ancestry-of-Origin Loci in Hispanic and Latino Individuals in the United States. J Am Soc Nephrol. 2022;33(1):77-87.
35. Wang H, Cade BE, Sofer T, Sands SA, Chen H, Browning SR, et al. Admixture mapping identifies novel loci for obstructive sleep apnea in Hispanic/Latino Americans. Hum Mol Genet. 2019;28(4):675-87.
36. Moon JY, Wang T, Sofer T, North KE, Isasi CR, Cai J, et al. Objectively Measured Physical Activity, Sedentary Behavior, and Genetic Predisposition to Obesity in U.S. Hispanics/Latinos: Results From the Hispanic Community Health Study/Study of Latinos (HCHS/SOL). Diabetes. 2017;66(12):3001-12.
37. Sofer T, Moon JY, Isasi CR, Qi Q, Shah NA, Kaplan RC, et al. Relationship of genetic determinants of height with cardiometabolic and pulmonary traits in the Hispanic Community Health Study/Study of Latinos. Int J Epidemiol. 2018;47(6):2059-69.
38. McArdle CE, Bokhari H, Rodell CC, Buchanan V, Preudhomme LK, Isasi CR, et al. Findings from the Hispanic Community Health Study/Study of Latinos on the Importance of Sociocultural Environmental Interactors: Polygenic Risk Score-by-Immigration and Dietary Interactions. Front Genet. 2021;12:720750.
39. Isasi CR, Moon JY, Gallo LC, Qi Q, Wang T, Sotres-Alvarez D, et al. Chronic Stress, Genetic Risk, and Obesity in US Hispanic/Latinos: Results From the Hispanic Community Health Study/Study of Latinos. Psychosom Med. 2022;84(7):822-7.
40. Grinde KE, Qi Q, Thornton TA, Liu S, Shadyab AH, Chan KHK, et al. Generalizing polygenic risk scores from Europeans to Hispanics/Latinos. Genet Epidemiol. 2019;43(1):50-62.
41. Spear ML, Diaz-Papkovich A, Ziv E, Yracheta JM, Gravel S, Torgerson DG, et al. Recent shifts in the genomic ancestry of Mexican Americans may alter the genetic architecture of biomedical traits. Elife. 2020;9.
42. Zhang Y, Elgart M, Kurniansyah N, Spitzer BW, Wang H, Kim D, et al. Genetic determinants of cardiometabolic and pulmonary phenotypes and obstructive sleep apnoea in HCHS/SOL. EBioMedicine. 2022;84:104288.
43. Hu Y, Graff M, Haessler J, Buyske S, Bien SA, Tao R, et al. Minority-centric meta-analyses of blood lipid levels identify novel loci in the Population Architecture using Genomics and Epidemiology (PAGE) study. PLoS Genet. 2020;16(3):e1008684.
44. Kurniansyah N, Goodman MO, Kelly TN, Elfassy T, Wiggins KL, Bis JC, et al. A multi-ethnic polygenic risk score is associated with hypertension prevalence and progression throughout adulthood. Nat Commun. 2022;13(1):3549.
45. Guo Y, Moon JY, Laurie CC, North KE, Sanchez-Johnsen LAP, Davis S, et al. Genetic predisposition to obesity is associated with asthma in US Hispanics/Latinos: Results from the Hispanic Community Health Study/Study of Latinos. Allergy. 2018;73(7):1547-50.
46. Hu X, Qiao D, Kim W, Moll M, Balte PP, Lange LA, et al. Polygenic transcriptome risk scores for COPD and lung function improve cross-ethnic portability of prediction in the NHLBI TOPMed program. Am J Hum Genet. 2022;109(5):857-70.
47. Fernandez-Rhodes L, Gong J, Haessler J, Franceschini N, Graff M, Nishimura KK, et al. Trans-ethnic fine-mapping of genetic loci for body mass index in the diverse ancestral

populations of the Population Architecture using Genomics and Epidemiology (PAGE) Study reveals evidence for multiple signals at established loci. Hum Genet. 2017;136(6):771-800.
48. Fernandez-Rhodes L, Graff M, Buchanan VL, Justice AE, Highland HM, Guo X, et al. Ancestral diversity improves discovery and fine-mapping of genetic loci for anthropometric traits-The Hispanic/Latino Anthropometry Consortium. HGG Adv. 2022;3(2):100099.
49. Zubair N, Graff M, Luis Ambite J, Bush WS, Kichaev G, Lu Y, et al. Fine-mapping of lipid regions in global populations discovers ethnic-specific signals and refines previously identified lipid loci. Hum Mol Genet. 2016;25(24):5500-12.
50. Franceschini N, Carty CL, Lu Y, Tao R, Sung YJ, Manichaikul A, et al. Variant Discovery and Fine Mapping of Genetic Loci Associated with Blood Pressure Traits in Hispanics and African Americans. PLoS One. 2016;11(10):e0164132.
51. Bien SA, Pankow JS, Haessler J, Lu Y, Pankratz N, Rohde RR, et al. Transethnic insight into the genetics of glycaemic traits: fine-mapping results from the Population Architecture using Genomics and Epidemiology (PAGE) consortium. Diabetologia. 2017;60(12):2384-98.
52. Kocarnik JM, Richard M, Graff M, Haessler J, Bien S, Carlson C, et al. Discovery, fine-mapping, and conditional analyses of genetic variants associated with C-reactive protein in multiethnic populations using the Metabochip in the Population Architecture using Genomics and Epidemiology (PAGE) study. Hum Mol Genet. 2018;27(16):2940-53.
53. Downie CG, Dimos SF, Bien SA, Hu Y, Darst BF, Polfus LM, et al. Multi-ethnic GWAS and fine-mapping of glycaemic traits identify novel loci in the PAGE Study. Diabetologia. 2022;65(3):477-89.
54. Magi R, Horikoshi M, Sofer T, Mahajan A, Kitajima H, Franceschini N, et al. Trans-ethnic meta-regression of genome-wide association studies accounting for ancestry increases power for discovery and improves fine-mapping resolution. Hum Mol Genet. 2017;26(18):3639-50.
55. Fernandez-Rhodes L, Malinowski JR, Wang Y, Tao R, Pankratz N, Jeff JM, et al. The genetic underpinnings of variation in ages at menarche and natural menopause among women from the multi-ethnic Population Architecture using Genomics and Epidemiology (PAGE) Study: A trans-ethnic meta-analysis. PLoS One. 2018;13(7):e0200486.
56. Jo Hodonsky C, Schurmann C, Schick UM, Kocarnik J, Tao R, van Rooij FJ, et al. Generalization and fine mapping of red blood cell trait genetic associations to multi-ethnic populations: The PAGE Study. Am J Hematol. 2018.
57. Wojcik GL, Graff M, Nishimura KK, Tao R, Haessler J, Gignoux CR, et al. Genetic analyses of diverse populations improves discovery for complex traits. Nature. 2019;570(7762):514-8.
58. Scannell Bryan M, Sofer T, Mossavar-Rahmani Y, Thyagarajan B, Zeng D, Daviglus ML, et al. Mendelian randomization of inorganic arsenic metabolism as a risk factor for hypertension- and diabetes-related traits among adults in the Hispanic Community Health Study/Study of Latinos (HCHS/SOL) cohort. Int J Epidemiol. 2019;48(3):876-86.
59. Scannell Bryan M, Sofer T, Afshar M, Mossavar-Rahmani Y, Hosgood HD, Punjabi NM, et al. Mendelian randomization analysis of arsenic metabolism and pulmonary function within the Hispanic Community Health Study/Study of Latinos. Sci Rep. 2021;11(1):13470.
60. Lee Y, Chen H, Chen W, Qi Q, Afshar M, Cai J, et al. Metabolomic Associations of Asthma in the Hispanic Community Health Study/Study of Latinos. Metabolites. 2022;12(4).
61. Granot-Hershkovitz E, He S, Bressler J, Yu B, Tarraf W, Rebholz CM, et al. Plasma metabolites associated with cognitive function across race/ethnicities affirming the importance of healthy nutrition. Alzheimers Dement. 2023;19(4):1331-42.
62. Noordam R, Sitlani CM, Avery CL, Stewart JD, Gogarten SM, Wiggins KL, et al. A genome-wide interaction analysis of tricyclic/tetracyclic antidepressants and RR and QT intervals: a pharmacogenomics study from the Cohorts for Heart and Aging Research in Genomic Epidemiology (CHARGE) consortium. J Med Genet. 2017;54(5):313-23.

63. Preudhomme LK, Gellman MD, Franceschini N, Perreira KM, Fernandez-Rhodes LE, Gallo LC, et al. Genetic and stress influences on the prevalence of hypertension among hispanics/latinos in the hispanic community health study/study of latinos (HCHS/SOL). Blood Press. 2022;31(1):155-63.
64. Dunn EC, Wiste A, Radmanesh F, Almli LM, Gogarten SM, Sofer T, et al. Genome-Wide Association Study (Gwas) and Genome-Wide by Environment Interaction Study (Gweis) of Depressive Symptoms in African American and Hispanic/Latina Women. Depress Anxiety. 2016;33(4):265-80.
65. Luo K, Chen GC, Zhang Y, Moon JY, Xing J, Peters BA, et al. Variant of the lactase LCT gene explains association between milk intake and incident type 2 diabetes. Nat Metab. 2024;6(1):169-86.
66. Reynolds KM, Horimoto ARVR, Lin BM, Zhang Y, Kurniansyah N, Yu B, et al. Ancestry-driven metabolite variation provides insights into disease states in admixed populations. Genome Med. 2023;15(1):52.
67. Qi Q, Li J, Yu B, Moon JY, Chai JC, Merino J, et al. Host and gut microbial tryptophan metabolism and type 2 diabetes: an integrative analysis of host genetics, diet, gut microbiome and circulating metabolites in cohort studies. Gut. 2022;71(6):1095-105.
68. Granot-Hershkovitz E, Spitzer B, Yang Y, Tarraf W, Yu B, Boerwinkle E, et al. Genetic loci of beta-aminoisobutyric acid are associated with aging-related mild cognitive impairment. Transl Psychiatry. 2023;13(1):140.
69. Kurniansyah N, Wallace DA, Zhang Y, Yu B, Cade B, Wang H, et al. An integrated multi-omics analysis of sleep-disordered breathing traits implicates P2XR4 purinergic signaling. Commun Biol. 2023;6(1):125.
70. Han X, Lains I, Li J, Chen Y, Yu B, Qi Q, et al. Integrating genetics and metabolomics from multi-ethnic and multi-fluid data reveals putative mechanisms for age-related macular degeneration. Cell Rep Med. 2023;4(7):101085.
